# The middle domain of Hsp104 can ensure substrates are functional after processing

**DOI:** 10.1371/journal.pgen.1011424

**Published:** 2024-10-03

**Authors:** Hannah E. Buchholz, Jane E. Dorweiler, Sam Guereca, Brett T. Wisniewski, James Shorter, Anita L. Manogaran

**Affiliations:** 1 Department of Biological Sciences, Marquette University, Milwaukee, Wisconsin, United States of America; 2 Department of Biochemistry and Biophysics, Perelman School of Medicine, University of Pennsylvania, Philadelphia, Pennsylvania, United States of America; Georgia Institute of Technology, UNITED STATES OF AMERICA

## Abstract

Molecular chaperones play a central role in protein disaggregation. However, the molecular determinants that regulate this process are poorly understood. Hsp104 is an AAA+ ATPase that disassembles stress granules and amyloids in yeast through collaboration with Hsp70 and Hsp40. *In vitro* studies show that Hsp104 processes different types of protein aggregates by partially translocating or threading polypeptides through the central pore of the hexamer. However, it is unclear how Hsp104 processing influences client protein function *in vivo*. The middle domain (MD) of Hsp104 regulates ATPase activity and interactions with Hsp70. Here, we tested how MD variants, Hsp104^A503S^ and Hsp104^A503V^, process different protein aggregates. We establish that engineered MD variants fail to resolve stress granules but retain prion fragmentation activity required for prion propagation. Using the Sup35 prion protein, our *in vitro* and *in vivo* data indicate that the MD variants can disassemble Sup35 aggregates, but the disaggregated protein has reduced GTPase and translation termination activity. These results suggest that the middle domain can play a role in sensing certain substrates and plays an essential role in ensuring the processed protein is functional.

## Author summary

Protein misfolding and assembly can lead to the formation highly ordered aggregates called amyloid. Amyloid is associated with several proteinopathies, including Alzheimer’s and Parkinson’s disease. Hsp104, the major chaperone disaggregase in yeast, fragments amyloid associated with yeast prions by working in concert with Hsp70 and Hsp40 chaperones to extract monomers from the amyloid in a partial threading mechanism. Engineered variants within the middle domain (MD) of Hsp104, Hsp104^A503S^ and Hsp104^A503V^, effectively disaggregate and reduce toxicity associated with human aggregating proteins. However, how the middle domain influences protein processing is not clearly defined. Our *in vitro* and *in vivo* data suggest that the MD can help ensure that disaggregated proteins are functional immediately after processing.

## Introduction

Molecular chaperones are critical players in protein quality control, also known as protein homeostasis. Chaperones manage protein misfolding, limit further protein aggregation, and mediate the disaggregation of highly-ordered aggregates such as amyloid [1–5]. Additionally, chaperones play a critical role in disassembling biomolecular condensates, which are membraneless compartments that contain high concentrations of proteins and nucleic acids [6]. In the case of stress granules, chaperones are critical for condensate disassembly after the removal of transient environmental stress [7–9]. However, upon aging, decline of the protein quality control network can lead to the accumulation of protein aggregates that are associated with disease [4,10,11]. Studying how molecular chaperones process substrates during disaggregation may provide important therapeutic directions against these diseases.

Hsp104 is a hexameric AAA+ ATPase molecular chaperone. While not found in metazoans, the study of Hsp104 has greatly advanced our understanding of how both biomolecular condensates and amyloids are disassembled. Stress granules form in response to transient heat or chemical stress. Upon stress removal in yeast, Hsp104 rapidly disassembles these granules [12–14], likely by partially translocating client proteins for disaggregation [15]. Working in concert with Hsp70 and Hsp40, Hsp104 also drives propagation of higher-ordered amyloids called yeast prions. The eukaryotic release factor 3 in yeast, Sup35, is an essential GTPase required for translation termination. In addition to functions in translation, Sup35 can take on an alternative, self-perpetuating aggregated state called the [*PSI*^*+*^] prion. Hsp104 is required for propagation of [*PSI*^+^] by fragmenting the protein aggregate into transmissible particles that are passed to daughter cells [16–22]. Similar to stress granules, it has been proposed that Hsp104 also employs partial threading to fragment prion aggregates [23–26]. Partial threading means that only a portion of the substrate polypeptide gets translocated across the Hsp104 pore, and not the entire polypeptide. The charged middle domain of Sup35 is involved in the interaction with Hsp104, and is thought to stimulate Hsp104 ATPase activity [25,27,28]. Hsp104 activity disrupts contacts between Sup35 monomers thereby translocating Sup35 in a stepwise manner to release a partially-folded monomer [24,25,29]. Due the variety of Hsp104 substrates, from stress granules to prions, it is possible that Hsp104 displays functional plasticity based on the needs of the client [12].

The middle domain (MD) of Hsp104 influences protein function [30]. Located between two nucleotide-binding domains and physically positioned on the outside of the Hsp104 hexamer, the middle domain coordinates ATP hydrolysis function, interaction with Hsp70, and plays a role in prion propagation and thermotolerance [30–34]. Structural studies have shown that a region within the middle domain, motif-2, links neighboring ATPase units within the hexamer [34,35]. This motif has been suggested to bind to Hsp70, which activates the hexamer to mediate the binding to substrates [36–38]. Degenerate mutations of the A503 residue within motif-2 result in enhanced ATPase activity and ability to mitigate toxicity of neurodegenerative disease proteins in yeast (e.g., TDP-43, FUS, and α-synuclein) and *C*. *elegans*, and thus are considered potentiated [39]. For example, Hsp104^A503V^ not only has higher ATPase activity but can efficiently reactivate denatured luciferase trapped in aggregates independently of Hsp70 *in vitro* [39]. The increased ATP hydrolysis and putative co-chaperone independence possibly contribute to these variants more effectively disaggregating and alleviating toxicity of exogenous human aggregating peptides associated with amyotrophic lateral sclerosis (ALS, i.e., TDP-43 and FUS) and Parkinson’s Disease (i.e., α-synuclein) [39], as well as remodeling aggregates associated with systemic Transthyretin amyloidosis *in vivo* [40]. Interestingly, opposite effects are observed with native yeast prions. The Chernoff lab found that high overexpression of Hsp104^A503V^ is toxic in the presence of [*PSI*^+^] [41], suggesting that the Hsp104 MD may have functions beyond Hsp70 binding and ATPase function, and may contribute to substrate processing.

To gain further insight into Hsp104 MD function, we assessed how Hsp104^A503S^ and Hsp104^A503V^ MD mutations process different yeast protein aggregates, such as stress granules and yeast prions. While these mutations in the middle domain affect the ability of Hsp104 to recognize and/or process stress granule substrates, they do not influence the fragmentation activity required for the propagation of yeast prions. However, our *in vivo* and *in vitro* data suggest that while prion substrates are processed in these variants, the released protein is likely non-functional at least initially. These data suggest that the middle domain of Hsp104 may help control how substrates are processed by the central pore to ensure functional activity *in vivo*.

## Results

### Hsp104^A503S^ and Hsp104^A503V^ exhibit reduced stress granule disassembly

To test how the MD mutations impact long-term and short-term temperature stress, we expressed wildtype Hsp104 (Hsp104^WT^) and two MD variants, Hsp104^A503S^ and Hsp104^A503V^. These genes were driven by the native Hsp104 promoter, which contains a heat shock element, and supplied on a centromeric plasmid [39,42]. We also used an empty vector (EV) control, which only contained the native Hsp104 promoter (HSE). Since centromeric plasmids are considered low copy, rather than single copy vectors [43], plasmid derived expression from native promoters usually result in elevated protein levels compared to integrated endogenous promoters. Introduction of plasmids into wildtype strains did not exhibit toxicity (S1A and S1B Fig), or impact endogenous Hsp104 steady state levels (S1C Fig). Hsp104 steady state levels from these three plasmids in wildtype cells (which contain a genomic copy of Hsp104) are held at approximately 4-fold over endogenous Hsp104 steady state levels (S1D and S1E Fig) [40]. Therefore, we refer to plasmids driven by the Hsp104 promoter as having ‘moderate overexpression’ when expressed in wildtype cells compared to galactose promoters that generally have ‘high overexpression.’ We found that these variants did not show any significant growth defects under chronic heat stress compared to controls (S2A Fig), however, Hsp104^A503V^ showed a very mild growth deficiency in wildtype cells at temperatures above 32°C (S2A Fig).

Next, we asked whether these plasmids have an effect on chronic stress in the absence of endogenous *HSP104*. Steady state levels of Hsp104^A503S^ and Hsp104^A503V^ expressed from these plasmids appeared to be comparable in *hsp104Δ* strains (S1F and S1G Fig) and had no apparent defects on growth compared to Hsp104^WT^ in chronic heat stress (S2B Fig). Mild pre-heat treatment helps cells survive elevated temperatures, and Hsp104 is required for this thermotolerance [44]. We found that both moderate overexpression of Hsp104^WT^ and Hsp104^A503S^ allowed for survival in thermotolerance assays, but Hsp104^A503V^ did not, as previously observed [S2C Fig; 42]. These data suggest that Hsp104^A503S^ and Hsp104^A503V^ behave differently in the context of these heat shock treatments.

**Fig 1:**
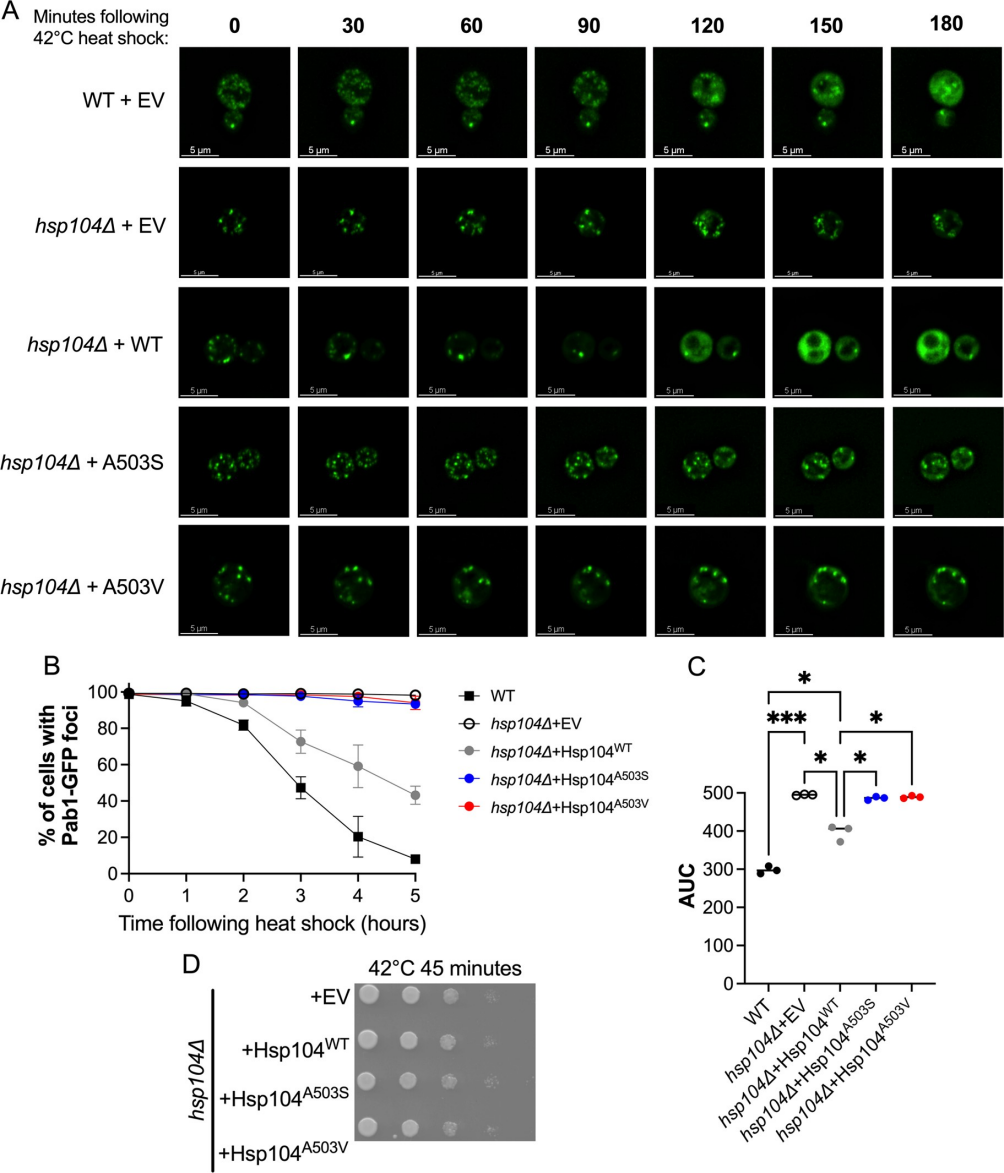
Hsp104^A503S^ and Hsp104^A503V^ exhibit reduced stress granule disassembly as the only source of Hsp104. A) WT and *hsp104Δ* strains, transformed with a plasmid containing the stress granule marker, poly-A binding protein, fused to GFP (Pab1-GFP) and the indicated HSE plasmids. Strains were heat shocked at 42°C for 45 minutes. Shown are representative cells from indicated strains undergoing stress granule disassembly following acute heat stress. All images were taken at the same magnification (630X) and scale bars are all equivalent to 5μm. B) Images were taken at the indicated time points following heat shock, and the number of cells with Pab1-GFP foci were quantified. C) Area under the curve (AUC) was quantified from B to determine statistical differences between strains. Analysis using Brown-Forsythe ANOVA with a Games-Howell’s multiple comparisons test showed the indicated significance bwteen strains (*p>0.03; ***p≤0.0009). D) *hsp104Δ* strains, as in A, were subjected to 42°C heat shock for 45 minutes and plated on SD-Ura.

Hsp104 disassembles heat-induced stress granules, which consist of both RNA and protein. These components include translation initiation factors, RNA-binding proteins, intrinsically disordered proteins and mRNAs [45]. Since the role of the MD of Hsp104 in stress granule disassembly is uncharacterized, we introduced the two MD variants as the only source of Hsp104 and tested for stress granule resolution. Using 3D time-lapse imaging and a poly-A binding protein fused to GFP (Pab1-GFP) to track stress granules, Pab1-GFP foci began to resolve within two hours of recovery from heat shock in wildtype cells. As expected, recovery was dramatically slower in *hsp104Δ* cells (Fig 1A and 1B), consistent with previous findings that Hsp104 plays an important role in stress granule disassembly [8,9]. Introduction of Hsp104^WT^ into *hsp104Δ* strains partially restored recovery to wildtype levels (Fig 1A and 1B). However, *hsp104Δ* strains expressing either Hsp104^A503S^ or Hsp104^A503V^ failed to resolve Pab1-GFP foci following 5 hours of recovery (Fig 1A and 1B). To determine whether stress granule recovery was statistically different between strains, we quantified these data by calculating the area under the curve (AUC). Using the AUC of WT, *hsp104Δ+*EV, and *hsp104Δ*+Hsp104^WT^, we confirm that Hsp104^WT^ does not restore recovery to wildtype conditions (Fig 1C). However, Hsp104^A503S^ and Hsp104^A503V^ have significantly different AUC values than Hsp104^WT^ (Fig 1C), despite exhibiting similar steady-state levels in our system (S1F and S1G Fig). Furthermore, the similarity of AUC values between *hsp104Δ* and Hsp104^A503S^ or Hsp104^A503V^, suggest that these variants have little impact on recovery (Fig 1C).

It has recently been reported that Hsp104^A503V^ as the sole source of Hsp104 in the BY4741 genetic background exhibited only a slight delay in the disassembly of heat-induced aggregates compared to wildtype [46]. We believe Hsp104^A503V^ exhibits a more pronounced defect in stress granule disassembly in our 74D-694 strain because of differences between genetic backgrounds and markers for heat-induced protein aggregation (FlucSM-GFP vs. Pab1-GFP). The reduced ability of Hsp104^A503S^ and Hsp104^A503V^ to disassemble stress granules suggests the A503 residue of Hsp104 plays a role in the ability of Hsp104 to either recognize the condensate or functionally disassemble granules following acute heat stress. It is important to note that the short-term stress treatment of 42°C for 45 minutes, used in the study of stress granules in this strain, was not associated with cell toxicity (Fig 1D).

### High overexpression toxicity is both mutant and prion variant dependent

**Fig 2:**
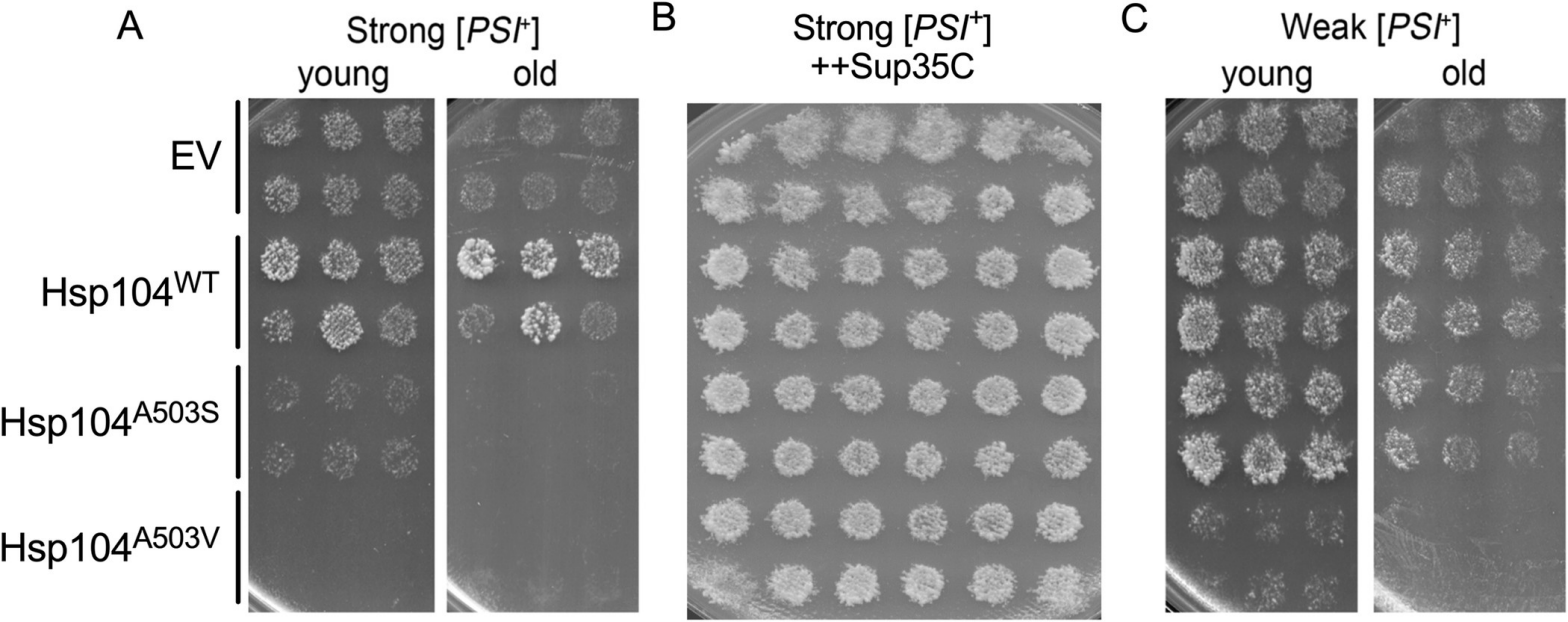
Hsp104^A503S^ is toxic in strong [*PSI*^*+*^] but not weak [*PSI*^*+*^]. A) *Left* Galactose-inducible Hsp104 plasmids were transformed into a wildtype strong [*PSI*^*+*^][*pin*^-^] strain. *Left*, six transformants of each indicated strain were first pronged onto media containing 2% dextrose media lacking uracil and grown for 2 days (young). Strains were then velveted onto 2% galactose media lacking uracil to induce plasmid expression. The image of toxicity shown in the figure is observed after three passages on 2% galactose media. *Right* The original transformants plated on 2% dextrose were incubated for one week (old) before being velveted onto 2% galactose media lacking uracil to induce plasmid expression. The image of toxicity shown is after two passages on 2% galactose media. B) Galactose-inducible Hsp104 plasmids as well as a galactose-inducible Sup35C plasmid were transformed into a wildtype strong [*PSI*^*+*^][*pin*^-^] strain. Twelve transformants of each strain were pronged onto media containing 2% dextrose media lacking uracil and tryptophan and grown for two days, then velveted onto 2% galactose media lacking uracil and tryptophan to induce plasmid expression. Viability shown is after two passages on 2% galactose media. C) Same as A, but in a wildtype weak [*PSI*^*+*^][*pin*^-^] strain.

Prion variants, such as strong [*PSI*^+^] or weak [*PSI*^+^] arise due to conformational differences in the protein aggregate and soluble pool of Sup35. Strong [*PSI*^+^] has smaller aggregates, less soluble Sup35, and is resistant to transient heat shock, whereas weak [*PSI*^+^] has larger aggregates, more soluble Sup35, and is sensitive to transient heat shock [47–50]. Previous work showed that high, prolonged overexpression of Hsp104^A503V^ (from a galactose promoter) in wildtype [*PSI*^+^] cells resulted in toxicity [41]. In this scenario, it is envisioned that the high overexpression leads to the formation of both Hsp104^A503V^ homohexamers, as well as WT/Hsp104^A503V^ heterohexamers [12, 51]. This toxicity was mitigated by the introduction of the Sup35 C-terminal domain, which likely restores translational termination function. It was proposed that Hsp104^A503V^ may play a role in sequestering the small soluble pool of Sup35 into the aggregate [41]. Here, we tested whether Hsp104^A503S^ exhibited similar toxicity issues. In the presence of strong [*PSI*^*+*^], Hsp104^A503S^ strains freshly plated onto galactose media grew better than Hsp104^A503V^ strains (Fig 2A, young). Upon chronological aging where strains were incubated for ten days at room temperature on glucose, prior to being plated onto galactose media, both Hsp104^A503S^ and Hsp104^A503V^ were toxic (Fig 2A, old). Similar to previous results, the introduction of the C-terminal domain of Sup35 prior to growth on galactose rescued toxicity in all strong [*PSI*^+^] strains (Fig 2B), suggesting that Hsp104^A503S^ and Hsp104^A503V^ elicit similar phenotypes in the presence of strong [*PSI*^+^]. However, it is important to note that it is unclear whether these variants cause soluble Sup35 protein to rejoin the aggregate as previously suggested or simply renders the disassembled Sup35 non-functional. Interestingly, we find that Hsp104^A503V^, but not Hsp104^A503S^, is toxic in the presence of weak [*PSI*^*+*^], suggesting that the mutations at A503 may confer different substrate conformation recognition (Fig 2C).

### Moderate overexpression of Hsp104^A503V^ does not cure weak [*PSI*^+^]

**Fig 3:**
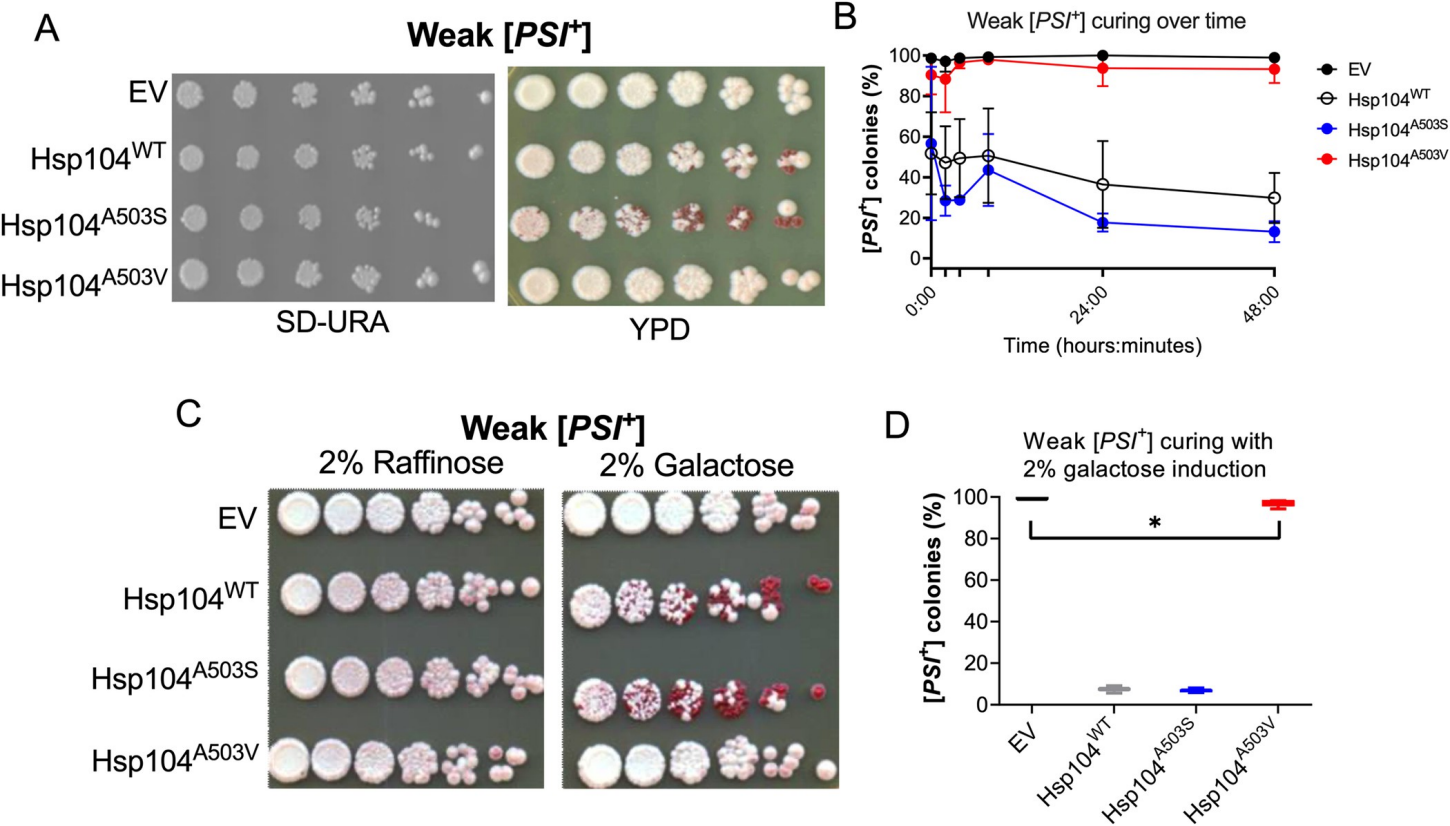
Hsp104^A503S^ is sufficient to cure weak [*PSI*^*+*^] similarly to Hsp104^WT^. A) Wildtype weak [*PSI*^*+*^][*pin*^-^] strains with indicated HSE plasmids were plated on SD-Ura to assess toxicity and on YPD to assess [*PSI*^*+*^] curing by a colony color assay. Shown is a representative of three trials. B) Fresh transformants of a weak [*PSI*^*+*^] strain with indicated plasmids were grown in liquid SD-Ura media and plated for single colony onto YPD at the indicated time points. Curing was determined by colony color 7 days after the cells were plated. Three trials were conducted, 50–150 colonies were assessed for each trial. Two-way ANOVA with Tukey’s multiple comparisons tests detected significant differences between Hsp104^WT^ and Hsp104^A503V^ at 48 hours p = 0.0118. C) Weak [*PSI*^*+*^] strains with indicated galactose-inducible plasmids were grown in 2% raffinose or 2% galactose media lacking uracil to an OD_600_ of 0.8–1.0 (approximately 20 hours) and plated on YPD to visually assess [*PSI*^+^] curing by colony color. The labeling indicates whether the strains were initially grown in either raffinose (no induction) or galactose (induction) prior to plating on YPD to assess color. E) Strains in C were also plated for single colony and scored for the presence of [*PSI*^+^]. At least three trials were conducted and approximately 800–1,500 colonies were assessed per trial. (*p = 0.027 t-test with Welch’s correction).

To determine whether the toxicity observed in Fig 2 was a byproduct of high overexpression from galactose promoters, we looked at how moderate overexpression of these variants from HSE promoters (described in S1D and S2A Figs) impacted toxicity and [*PSI*^+^] propagation. There was no noticeable toxicity observed with moderate overexpression (Figs 3A and S3; SD-Ura). Since there was no toxicity observed, we next asked whether moderate overexpression of these variant proteins results in prion loss.

It is well established that high overexpression of Hsp104 results in prion loss or “curing” of strong and weak variants of [*PSI*^+^] [16, 47]. We found that moderate overexpression of Hsp104^WT^, Hsp104^A503S^ or Hsp104^A503V^had no impact on strong [*PSI*^+^], as assayed by colony color (see materials and methods; S3 Fig). In contrast, weak [*PSI*^*+*^] was partially cured by Hsp104^WT^ and Hsp104^A503S^ but not by Hsp104^A503V^ (Fig 3A), suggesting that Hsp104^A503S^ and Hsp104^A503V^ may not be functionally similar in the context of weak [*PSI*^+^] curing. To monitor prion loss over time, weak [*PSI*^*+*^] cultures were grown and plated for single colonies at different points over a 48-hour period. While curing effects were immediately observed in Hsp104^WT^ and Hsp104^A503S^ strains, the percentage of [*PSI*^+^] colonies in Hsp104^WT^ and Hsp104^A503S^ was dramatically reduced with average remaining amount of [*PSI*^+^] colonies of 29.8% and 13.2% respectively by 48 hours (Fig 3B). 99.0% of EV colonies remained [*PSI*^+^] throughout the time course, which is similar to low level of spontaneous prion loss observed in other studies [50, 52]. 93.3% of Hsp104^A503V^ colonies were [*PSI*^+^] by 48 hours, suggesting that Hsp104^A503V^ may have subtle curing ability, despite being expressed at similar levels to Hsp104^WT^ in wildtype strains (S1D and S1E Fig). Taken together, the disparity in curing ability between Hsp104^A503S^ and Hsp104^A503V^ suggests that the A503 residue may play a role in modulating Hsp104 overexpression curing function.

To transiently control Hsp104 overexpression, weak [*PSI*^+^] strains carrying galactose inducible Hsp104 plasmids were grown transiently in liquid galactose media to late log phase instead of repeated plating on galactose plates (Fig 2C). These strains were then plated on rich glucose media (YPD) to assess viability by growth and [*PSI*^+^] loss by colony color. Transient high overexpression did not result in toxicity, yet Hsp104^WT^ and Hsp104^A503S^ strains showed high curing rates, with less than 10% of colonies retaining [*PSI*^+^] (Fig 3C and 3D). Lower galactose induced expression of Hsp104^WT^ and Hsp104^A503S^ confirmed that these two strains had similar curing efficiencies (S4A and S4B Fig). While transient galactose induction of Hsp104^A503V^ resulted in low curing similar to Fig 3A and 3B, these experiments show that curing was slightly higher in Hsp104^A503V^ than EV strains (Fig 3D).

### Hsp104^A503S^ and Hsp104^A503V^ are toxic in the presence of [*PSI*^+^] as the only source of Hsp104

**Fig 4:**
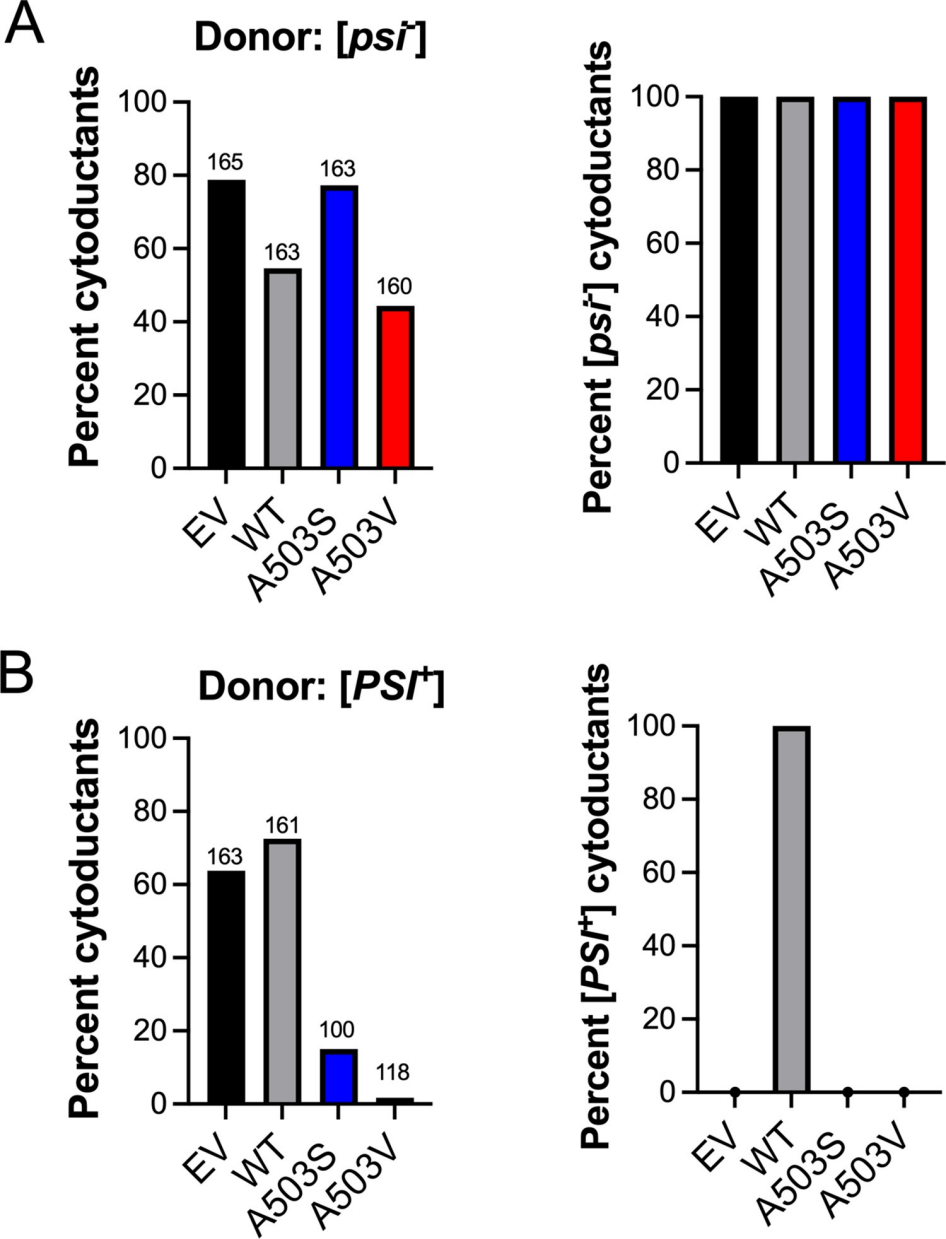
Hsp104^A503S^ or Hsp104^A503V^ as the only source of Hsp104 cannot maintain [*PSI*^*+*^]. A) *Left panel*, cytoduction was performed from a donor strain lacking prions ([*psi*^-^][*pin*^*-*^]) to a *hsp104Δ* recipient strain containing the indicated HSE plasmid. Definitive cytoductants were identified based upon acquisition of [*RHO*^*+*^] phenotype, and cytoductant selective markers. Numbers above bars represent the total sample number of potential cytoductants that were screened whereas the bar indicates the percentage of definitive cytoductants obtained. *Right panel*, definitive cytoductants were assayed for the [*PSI*^*+*^] state. B) *Left panel*, the percentage of definitive cytoductants obtained from a strong [*PSI*^*+*^] donor. *Right panel*, quantification of cytoductants that were able to retain [*PSI*^*+*^].

The experiments above were performed in the presence of endogenous Hsp104, suggesting that the Hsp104 hexamer in these experiments are likely composed of both variant and endogenous wildtype subunits, similar to previous *in vitro* studies [39,51]. To assess how these MD variants can maintain the [*PSI*^*+*^] prion as the only source of Hsp104, we utilized a process called cytoduction [53]. Cytoduction allows us to introduce [*PSI*^+^] in a *hsp104Δ* strain containing moderately overexpressed Hsp104 plasmids as the only source of Hsp104. To determine whether the variant Hsp104 proteins interfere with the cytoduction process, we first performed cytoduction in the absence of prions (see materials and methods). All plasmids were able to generate [*psi*^-^] cytoductants, where acquisition of mitochondrial DNA ([*RHO*^*+*^]) and presence of cytoductant specific markers confirmed the colony was a definitive cytoductant (Fig 4A, left). As expected, all the [*psi*^-^] cytoductants from each mating scheme remained [*psi*^-^] (Fig 4A, right), indicating that these plasmids do not interfere with cytoduction or result in the spontaneous formation of [*PSI*^+^].

To test whether Hsp104^WT^, Hsp104^A503S^, or Hsp104^A503V^ could maintain [*PSI*^*+*^] in a *hsp104Δ* background, we used strong [*PSI*^+^] donors since we observed that this variant could not be cured with this level of overexpression (S3 Fig). As expected, the EV and Hsp104^WT^ strain both produced cytoductants (Fig 4B, left) but only the Hsp104^WT^ was able to maintain [*PSI*^+^] (Fig 4B, right). In contrast, Hsp104^A503S^ and Hsp104^A503V^ had dramatically reduced cytoduction frequencies. Of the few Hsp104^A503S^ and Hsp104^A503V^ cytoductants that were obtained, none were unable to maintain [*PSI*^*+*^] (Fig 4B), suggesting that these Hsp104 MD variants may cause toxicity during the cytoduction process. These surprising Hsp104^A503S^ and Hsp104^A503V^ results are in stark contrast to the observations of Fig 3, where expression of these same plasmids in a wildtype background resulted in viability and [*PSI*^*+*^] maintenance. We suspect that the observed toxicity is due to high occupancy of the Hsp104 hexamer with variant subunits either when Hsp104 variants are highly expressed in wildtype cells (Fig 2) or when they are the only source of Hsp104 (Fig 4).

To determine whether the lack of Hsp104^A503S^ or Hsp104^A503V^ [*PSI*^*+*^] cytoductants was due to toxicity rather than an artifact of cytoduction, we turned to sporulation. [*PSI*^+^] exhibits non-Mendelian inheritance in which mating between [*PSI*^+^] and [*psi*^-^] wildtype haploid strains result in all viable spores containing [*PSI*^*+*^] [54]. Yet, sporulation of a [*PSI*^+^] *HSP104/ hsp104Δ* heterozygous diploid results in [*PSI*^+^] segregating only with *HSP104* spores [55,56]. To generate [*PSI*^*+*^] strains where the only source of Hsp104 was from the plasmid, wildtype strong [*PSI*^+^] strains were mated with *hsp104Δ* containing either EV, Hsp104^WT^, Hsp104^A503S^ or Hsp104^A503V^. Diploids were sporulated and subjected to random spore analysis. Selecting for *hsp104Δ* spores containing the plasmid, EV spores were viable but did not maintain [*PSI*^+^], whereas Hsp104^WT^ was able to maintain [*PSI*^+^] in approximately 73% of the spores (Table 1). Interestingly, while *hsp104Δ* spores containing Hsp104^A503S^ were recovered, none were [*PSI*^+^] (Tables 1 and S1). In stark contrast, Hsp104^A503V^ matings did not recover any viable *hsp104Δ* Hsp104^A503V^ spores in two independent experiments (Tables 1 and S1).

**Table 1:** Hsp104^A503S^ spores are viable but cannot maintain [*PSI*^+^], and Hsp104^A503V^ spores are not viable.

Strain	EV	Hsp104^WT^	Hsp104^A503S^	Hsp104^A503V^
Total spores obtained	105	85	287*	0
% [*PSI*^*+*^]	0	72.9	0	N/A
% [*psi*^*-*^]	100	27.1	100	N/A

Spores referred to in the table are *hsp104Δ* that contain the plasmid. Data represents the combined totals from two trials. See S1 Table for individual trial data. *Due to results from the first trial, we over-picked Hsp104^A503S^ colonies (See S1 Table).

Sporulation in yeast involves two meiotic events to generate four spores, called a tetrad. To investigate whether there were any sporulation issues, we monitored the formation of tetrads over time using DAPI staining, where diploids contain one nuclei and spores transitioning into tetrads have two to four nuclei over time. The number of tetrads formed over time was low from [*psi*^-^] *HSP104/ hsp104Δ* heterozygous diploids with plasmids, as determined by four DAPI stained nuclei per tetrad (See materials and methods; S5A and S5B Fig), compared to other genetic backgrounds such as W303, which exhibits 50% sporulation in eight days [57]. All strains showed an increase in irregular DAPI stained bodies that are different from tetrad staining (S5A and S5B Fig). While it is unclear why this strain produces cells with these irregular bodies, other studies suggest that irregular staining may be a result of aberrant nuclear divisions, issues within meiosis II, or improper sequestration factors that influence nuclear senescence during meiosis such as protein aggregates [58,59]

Tetrad formation was monitored in strong [*PSI*^*+*^] *HSP104/ hsp104Δ* heterozygous diploids. Higher percentages of tetrads were observed in diploids containing EV and Hsp104^WT^ plasmids in [*PSI*^+^] diploids compared to [*psi*^-^] diploids, suggesting that [*PSI*^*+*^] may provide an advantage in sporulation in this genetic background (S5C Fig). These observations are consistent with previous studies that have suggested the presence of [*PSI*^*+*^] can confer chemical resistance to spores by providing phenotypic plasticity [60–62]. Sporulation of heterozygous [*PSI*^*+*^] diploids containing Hsp104^A503S^ (7.4%) and Hsp104^A503V^ (6.3%) exhibited reduced tetrad formation at 10 days compared to EV (21.5%) and Hsp104^WT^ (26.8%) (S5C Fig). Interestingly, irregular DAPI staining was greater in Hsp104^A503S^ and Hsp104^A503V^ in the presence of [*PSI*^+^] (S5A and S5C Fig). The increase of irregular DAPI stained [*PSI*^+^] spores corresponded with a decrease in tetrads (S5C Fig), which could suggest that these mutants interfere with meiosis completion or interfere with aggregate maintenance and turnover at the end of meiosis [63]. However, it is important to note that only Hsp104^A503V^ fails to produce viable spores in the presence of [*PSI*^+^]. Again, this A503 residue may fine tune Hsp104 meiotic function in the presence of an aggregating protein.

### Middle domain variants maintain fragmentation activity and do not change the soluble pool of Rnq1

**Fig 5:**
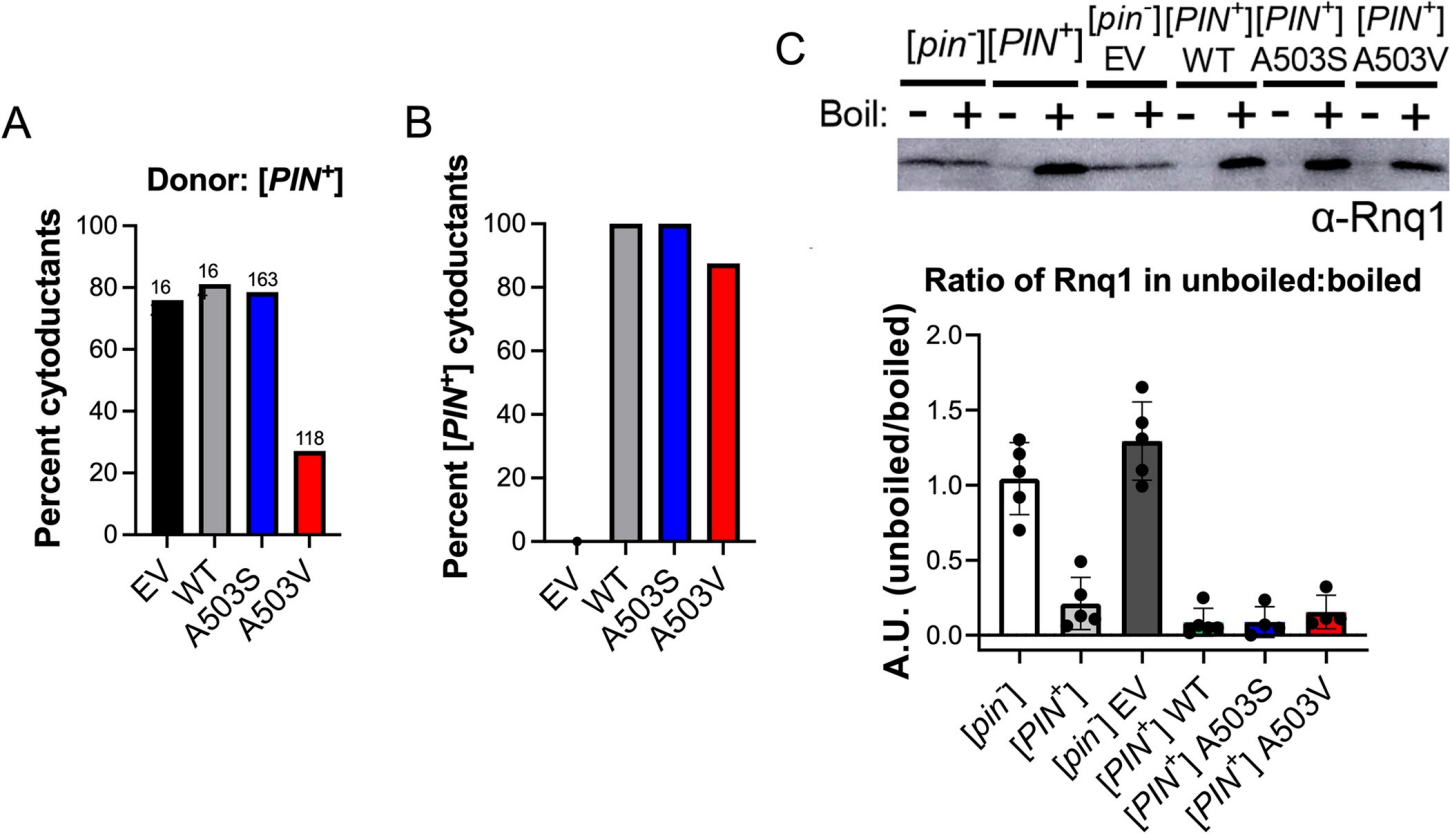
Hsp104^A503S^ and Hsp104^A503V^ retain [*PIN*^*+*^] prion fragmentation activity and do not change the soluble pool of Rnq1. A) The percentage of definitive cytoductants obtained from a [*psi*^*-*^] high [*PIN*^+^] donor. B) Quantification of cytoductants that were able to retain [*PIN*^*+*^]. C) Well trap assay used to determine the amount of SDS-sensitive soluble Rnq1 protein in the indicated [*PIN*^+^] cytoductants obtained from Fig 4C. Western blot (top) and quantification of four independent experiments (bottom) are shown.

To determine whether the toxicity observed above is a general prion phenomenon, or only applies to [*PSI*^+^], we performed similar cytoduction experiments using the prion form of the Rnq1 protein, [*PIN*^+^] [64–66]. [*PIN*^+^] shares many prion features with [*PSI*^*+*^], such as Hsp104 dependence and non-mendelian inheritance. However, it is different from [*PSI*^+^] because [*PIN*^+^] maintenance is not impacted by Hsp104 overexpression, and the *RNQ1* gene in not essential (compared to the essential *SUP35*). We obtained definitive [*PIN*^+^] cytoductants in the presence Hsp104^WT^, Hsp104^A503S^ and Hsp104^A503V^, albeit Hsp104^A503V^ exhibited a lower cytoduction frequency (Fig 5A). More importantly, these cytoductants were able to maintain [*PIN*^*+*^] (Fig 5B). These results not only indicate that toxicity of Hsp104^A503S^ and Hsp104^A503V^ is [*PSI*^+^] specific, but also indicate that these variants retain fragmentation activity since the [*PIN*^+^] prion can be propagated in these strains.

To determine whether the mutations reduce the amount of available soluble protein, we performed well trap assays. Well trap assays are used to detect SDS-sensitive, soluble protein, since larger SDS-resistant complexes are trapped in the well of the gel. Because we were unable to obtain viable [*PSI*^+^] cells in the presence of Hsp104^A503S^, and Hsp104^A503V^, we turned to [*PIN*^+^] cytoductants obtained in Fig 5A and 5B. There was no significant difference in the amount of soluble Rnq1 protein detected between Hsp104^WT^, Hsp104^A503S^, and Hsp104^A503V^ strains (Fig 5C), suggesting that Hsp104^A503S^, and Hsp104^A503V^ still fragments prion aggregates and the overall steady state of soluble pool of protein does not change.

### MD variants disassemble Sup35 prions but unfold the essential GTPase domain of Sup35 *in vitro*

**Fig 6:**
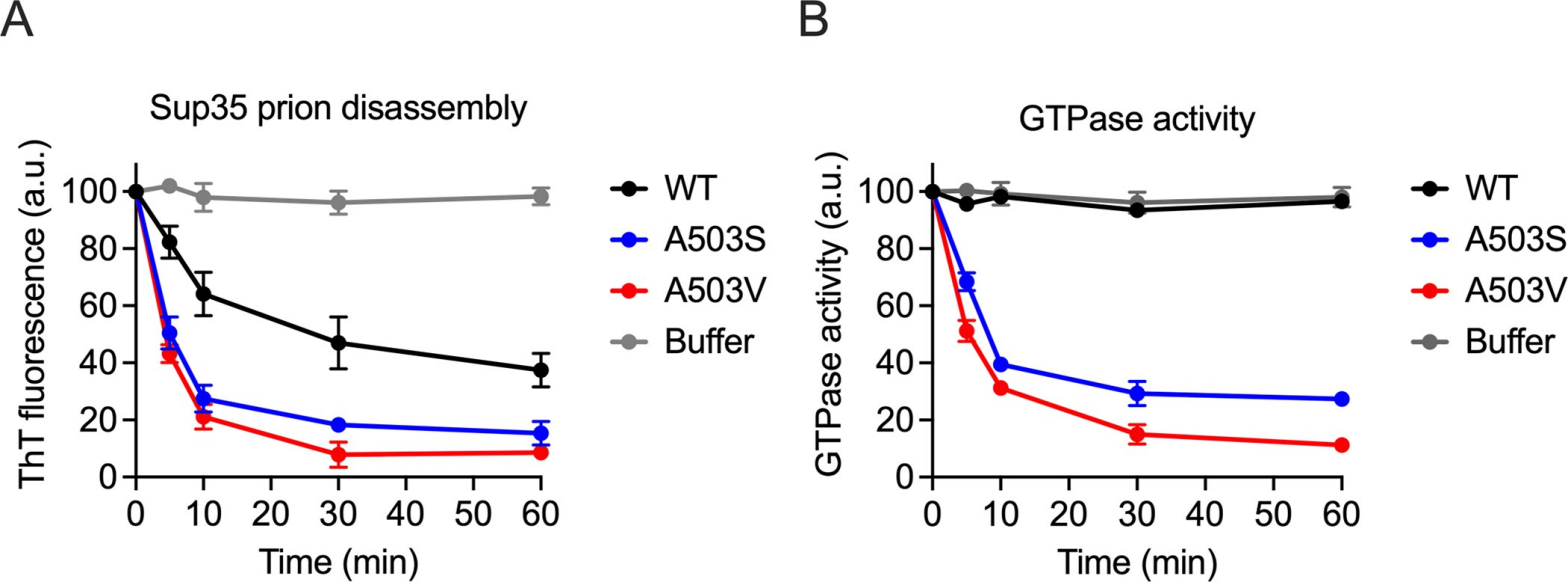
Hsp104^A503S^ and Hsp104^A503V^ disassemble Sup35 fibrils into non-functional protein *in vitro*. A) Preformed full-length Sup35 prions (1 μM monomer) were incubated with GroEL_trap_ (2 μM), Ssa1 (1μM), Sis1 (1μM), Sse1 (0.1μM), and either buffer, Hsp104^WT^, Hsp104^A503S^, or Hsp104^A503V^ (1μM) and monitored over 60 minutes. Sup35 fibril integrity was measured with Thioflavin-T (ThT) fluorescence. Values represent means±SEM (n = 3). B) The GTPase activity of full-length Sup35 from A was assessed by measuring the GTPase activity associated with the C-terminal domain of Sup35 over time. Values represent means±SEM (n = 3).

The data above indicate that the MD variants have fragmentation activity yet are toxic in the presence of [*PSI*^+^]. We speculated that [*PSI*^+^] toxicity could be due to how these variants process substrates. To test Sup35 processing, recombinant pre-formed Sup35 prions were combined with Ssa1 (Hsp70), Sis1 (Hsp40), Sse1 (Hsp110), GroEL_trap_, and either buffer, Hsp104^WT^, Hsp104^A503S^, or Hsp104^A503V^. In the absence of Hsp104, Sup35 prions retained ThT fluorescence, indicating that Hsp104 is essential for prion dissolution (Fig 6A). By contrast, ThT fluorescence declined upon addition of Hsp104^WT^, Hsp104^A503S^, or Hsp104^A503V^ (Fig 6A), indicating these aggregates were undergoing disassembly. Under these conditions, Hsp104^A503S^ and Hsp104^A503V^ were more effective than Hsp104^WT^ in disassembling Sup35 prions.

During [*PSI*^+^] fragmentation, Hsp104 has been proposed to partially translocate substrates through its central pore [25]. The partial translocation of Sup35 monomers allows for prion fragmentation, while retaining the essential Sup35 C-terminal GTPase domain intact and functional. Therefore, based on this model, *in vitro* disassembly of Sup35 monomers should result in functional Sup35 protein with GTPase activity. In the presence of Ssa1, Sis1, Sse1, GroEL_trap_, and Hsp104^WT^, disassembled Sup35 exhibited high GTPase activity over time indicating that these Sup35 monomers remain functional during the disaggregation process (Fig 6B). However, even though Sup35 fibril disassembly was robust in Hsp104^A503S^ or Hsp104^A503V^, Sup35 had very little GTPase activity. Under these conditions, our data suggest that while Hsp104^A503S^ and Hsp104^A503V^ disassemble prions, the resulting Sup35 monomers are non-functional, possibly due to complete unfolding of the Sup35 protein.

### MD variants increase stop codon readthrough in [*PSI*^+^] cells, possibly by reducing Sup35 translation termination activity

In wildtype [*PSI*^+^] cells, the amount of non-aggregated Sup35 is thought to be sufficient to maintain cell viability. However, it is also been shown that within the aggregate, the C-terminal domain of Sup35 is accessible and hydrolyzes GTP *in vitro* [67,68] and the [*PSI*^+^] aggregate contributes to translation termination activity *in vivo* [69]. Based on previous work and our data that Sup35 disassembled by MD variants has reduced GTPase activity *in vitro* (Fig 6), it is possible that the A503 residue of Hsp104 is critical for processing Sup35 from the aggregate to ensure its C-terminal domain is functional *in vivo*. Therefore, we tested whether MD variants reduced translational termination activity of Sup35 in [*PSI*^+^] strains.

**Fig 7:**
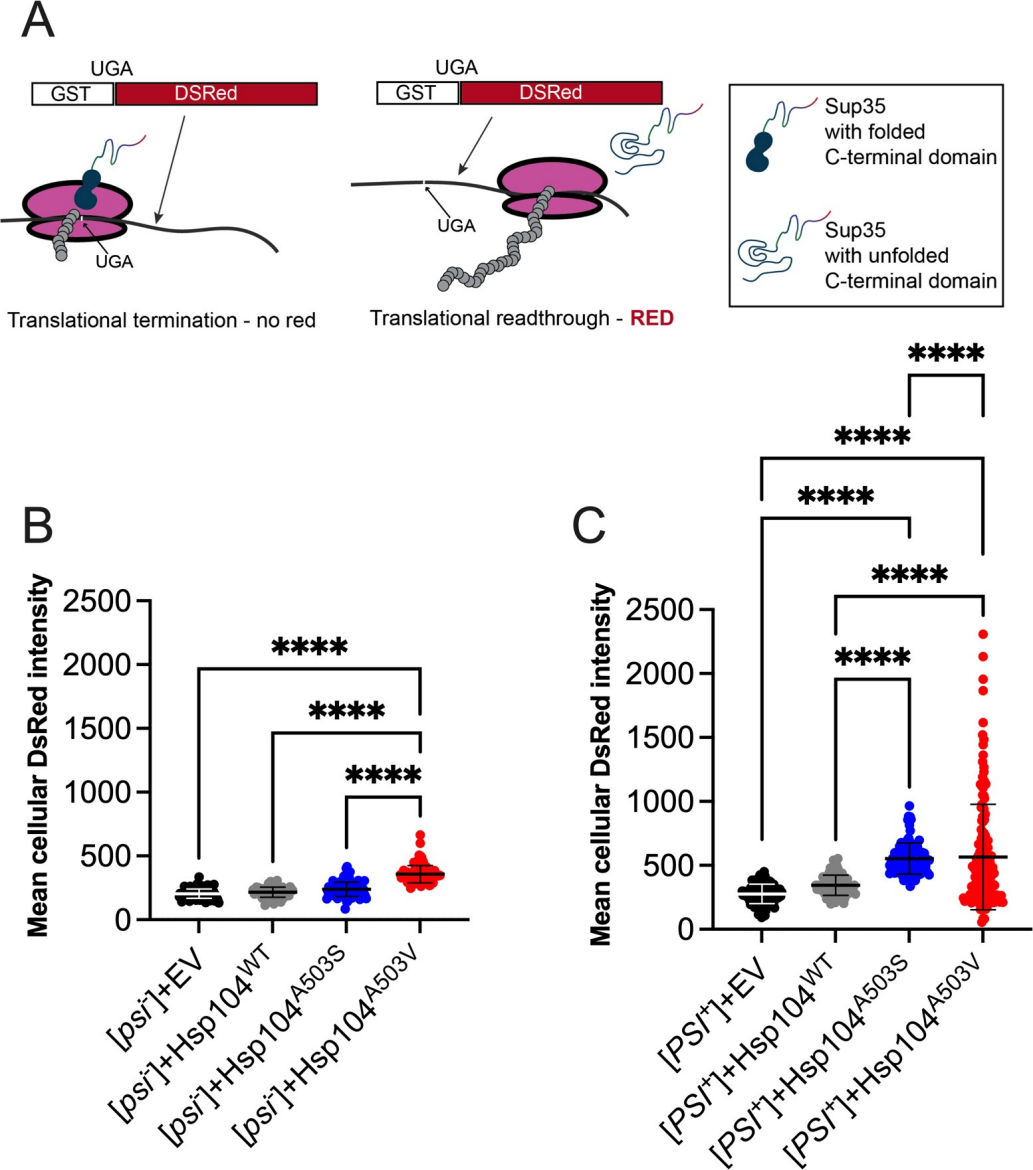
Hsp104^A503S^ and Hsp104^A503V^ increase stop codon readthrough. A) Schematic of indirectly assaying Sup35 function by quantifying fluorescent intensity of DsRed, which contains a UGA codon between GST and DsRed. In the presence of functional Sup35, translation termination at the UGA codon results in a truncated protein. In the presence of non-functional Sup35, translational readthrough of UGA will result in a GST-DsRed fusion protein. B) A [*psi*^*-*^][*pin*^-^] strain integrated with a GST(UGA)DsRed reporter was transformed with the indicated galactose-inducible Hsp104 variants. Strains were grown on galactose-containing media for two days to drive plasmid expression. Cells were picked directly from plates and subjected to fluorescent microscopy. Mean DsRed pixel intensity was measured in individual cells and normalized to the mean DsRed pixel intensity of the field background. Bars represent mean±SD Approximately 25–250 cells were counted for each of three trials for a minimum total of 275 cells. C) Same as in B, but in a [*PSI*^*+*^][*pin*^-^] strain. A Brown-Forsythe and Welch’s ANOVA test with Games-Howell’s multiple comparisons test was used to compare strains (****p≤0.0001).

Since we showed that [*PSI*^+^] cells are inviable when the variant is the sole source of Hsp104 (Fig 4), we expressed galactose inducible MD variants in wildtype [*PSI*^+^] strains used in Fig 2. While these strains exhibited toxicity after three subsequent passages on galactose containing media, cells were viable after one passage and could be used to assess Sup35 function. To assess function, this wildtype strain also contained an integrated GST(UGA)DsRed reporter, in which a premature UGA codon is placed within between the GST and DsRed (Fig 7A). In [*psi*^-^] cells, functional Sup35 is able to stop translation, leading to low DsRed fluorescence. In [*PSI*^+^] cells, because of the reduced amount of Sup35 available for translational termination, there is a higher likelihood that readthrough will occur resulting in a higher DsRed signal (Fig 7A) [70].

The GST(UGA)DsRed strains were transformed with the galactose-inducible Hsp104 wildtype or variant plasmids. Transformants were first grown on dextrose-containing media then passaged one time onto 2% galactose-containing media to drive high overexpression of Hsp104. After growth on galactose for two days, strains were visualized for DsRed fluorescence, and quantified using mean pixel intensity (Fig 7A).

In [*psi*^*-*^] strains, EV, Hsp104^WT^, and Hsp104^A503S^ had similar DsRed intensity (Fig 7B). Only Hsp104^A503V^ exhibited increased DsRed intensity (Fig 7B), suggesting that Hsp104^A503V^ influences the DsRed readout by itself or could unfold soluble Sup35. In [*PSI*^*+*^] strains, Hsp104^A503S^ or Hsp104^A503V^ both exhibited approximately a 2-fold increase in DsRed intensity compared to Hsp104^WT^ (Fig 7C). These data suggest that there is possibly less functional Sup35 is available for translation termination of GST(UGA)DsRed (Fig 7C). Interestingly, Hsp104^A503V^ exhibited more stop codon readthrough compared to Hsp104^A503S^, suggesting one accumulates more non-functional Sup35 than the other. It is important to note that stop codon readthrough was greater in all [*PSI*^*+*^] strains compared to equivalent [*psi*^*-*^] strains (S6 Fig), including Hsp104^A503V^, which showed an elevated [*psi*^*-*^] DsRed readout (Fig 7B). Taken together, the data presented in Figs 6 and 7 provide both *in vitro* and *in vivo* support that the influence of A503 in the MD on Hsp104 function may ensure that proteins are functional after Sup35 prion fragmentation.

## Discussion

Hsp104 fragments amyloids like yeast prions and disassembles stress granules [12–14,71,72], yet it is unclear how the disaggregation process influences protein function. Insight from *in vitro* studies of MD variants such as Hsp104^A503S^ and Hsp104^A503V^, which have elevated ATP hydrolysis activity and act independently of Hsp70 in luciferase reactivation assays [39], suggest the MD may control how substrates are processed. It is thought that Hsp104 can partially thread substrates from the larger aggregate rather than full translocation of a monomer through the Hsp104 central pore [15,23,25]. Our study implicates the MD in the ability of Hsp104 to recognize and process endogenous Hsp104 substrates *in vivo*. We find that as the only source of Hsp104, the MD variants, Hsp104^A503S^ and Hsp104^A503V^, fail to disassemble stress granules (Fig 1) and are toxic in the presence of [*PSI*^+^] (Figs 2 and 4 and Table 1). However, the MD variants retain prion fragmentation activity (Fig 5A and 5B), suggesting that the processing of [*PSI*^+^] and stress granules are different from wildtype Hsp104 activity. Importantly, our data lends evidence that the MD of Hsp104 plays a role in ensuring that processed proteins are released in a functional state (Figs 6 and 7).

### Middle domain of Hsp104 in recognition of amorphous aggregates vs. prions

*In vitro* evidence suggests that Hsp104 hexamer can switch between processing mechanisms based on the nature of the client or substrate protein. Amorphous aggregates can be resolved with as little as engagement as one subunit [12]; however, Hsp104^A503S^ and Hsp104^A503V^ fail to efficiently disassemble stress granules (Fig 1) despite retaining prion fragmentation activity (Fig 5). It is possible that requirements for processing prions and stress granules are different. While a prion is highly ordered, stress granules are less ordered and amorphous. The inability of MD variants to resolve stress granules could be due to lack of substrate or co-chaperone recognition. The middle domain of Hsp104 couples Hsp70 to amorphous aggregate disaggregation [38], and *in vitro* studies suggest that Hsp70 binding to Pab1-GFP condensates leads to an increase in Hsp104 binding and efficient condensate dispersal [15]. Although Hsp104^A503V^ resolves luciferase independently of Hsp70 *in vitro*, it is possible the MD variants are less efficient at recognizing Hsp70-bound stress granules *in vivo*. Conversely, while Hsp70 plays a role in prion propagation [73], the highly ordered conformation of the aggregate may allow MD variants to recognize Hsp70-bound prions and therefore mediate fragmentation. Based on these two results, it is possible that the middle domain plays a role in sensing certain substrates.

### Hsp104 hexamer subunit composition impacts amyloid processing in a variant-specific manner

We have established that moderate overexpression of Hsp104^A503S^ in the presence of endogenous Hsp104 results in weak [*PSI*^*+*^] curing. While Hsp104^A503S^ shows curing efficiencies similar to overexpression of Hsp104^WT^, Hsp104^A503V^ shows very little curing, suggesting the A503 residue may influence how substrates are processed (Fig 3A and 3B). Alternatively, this residue may dictate how Hsp104 hexamers are formed and how individual subunits engage and process diverse substrates. Amyloid fragmentation requires anywhere from 3–6 Hsp104 subunits depending on the specific amyloid conformation. For example, the engagement of subunits for fragmentation are different between [*PSI*^+^] variants with weak [*PSI*^+^] engaging more subunits than strong [*PSI*^+^] [12]. Furthermore, enhanced disaggregase activity by Hsp104^A503V^ requires that the variant needs to occupy approximately 2–3 subunits within the hexamer [51]. With moderate overexpression, it is likely that Hsp104 hexamers are composed of varying degrees of wildtype and variant subunits. In the case of Hsp104^A503S^, the influence of wildtype subunits could confer wildtype-like activity of variant subunits. In contrast, the number of Hsp104^A503V^ subunits within the hexamer could have an opposite effect on activity. If some heterohexamers are partially inactivated, there could be other heterohexamers with enhanced activity based on the number of variant subunits, thus resulting in a net zero difference in prion fragmentation and no observable curing. The amount of variant:wildtype Hsp104 subunits within a hexamer becomes critical when considering high, continuous overexpression of the MD variants. We show that high overexpression of Hsp104^A503S^ or Hsp104^A503V^, which likely involves the variant form occupying many or all subunits within the hexamer, is toxic in strong [*PSI*^+^] (Fig 2). Taken together, Hsp104^A503S^ and Hsp104^A503V^ may contribute differently to substrate recognition or processing compared to Hsp104^WT^, when the variants are the major source of Hsp104 subunits.

### A503 in the MD of Hsp104 is required to maintain function of the processed substrate, possibly through a partial threading mechanism

Studying Hsp104 heterohexamers has revealed that the MD influences prion processing in variant-specific manner based on the hexamer composition. When present as the only source of Hsp104 in [*PSI*^+^] strains, Hsp104^A503S^ and Hsp104^A503V^ are inviable (Fig 4B and Table 1). Sup35 is composed of a prion domain, a middle domain that binds to Hsp104, and a C-terminal GTPase domain that confers translation termination activity [25,27,47,74–76]. This GTPase domain is thought to retain its native fold and is accessible on the surface of the prion aggregate [67,77]. Both *in vitro* and *in vivo* evidence suggests that this GTPase domain also retains functional activity within the aggregate, suggesting that the prion aggregate can also participate in translation termination [Fig 6; 68,69].

**Fig 8:**
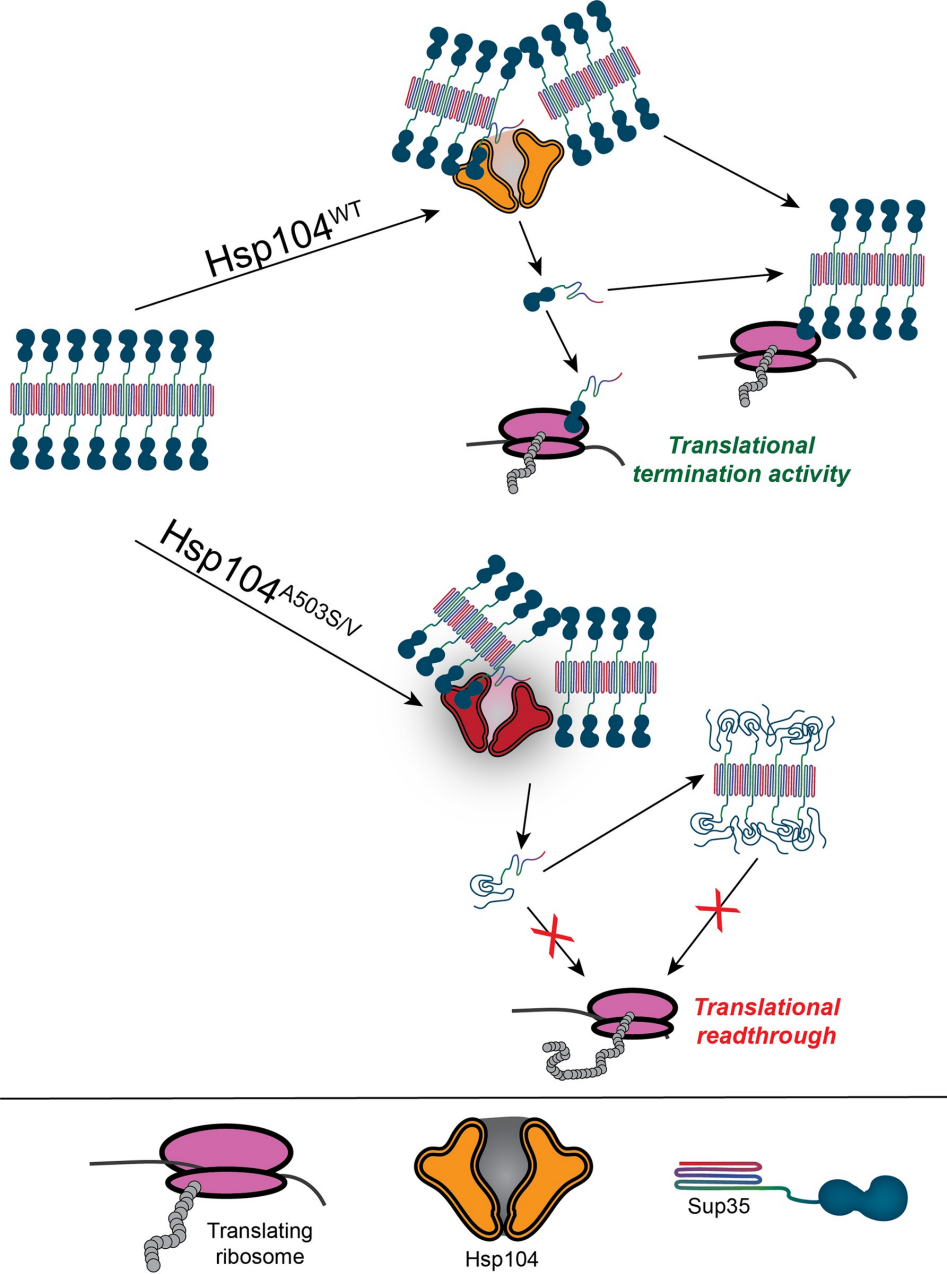
Contributions of the Hsp104 middle domain in prion processing. Sup35 contains a prion domain (shown as rainbow core), a linker thought to bind to Hsp104, and a functional GTPase C-terminal domain (shown as an attached blue shape). Within the prion aggregate, the C-terminal domain is accessible on the surface and retains translation termination activity. The A503 within the middle domain plays a role in ensuring that the extracted monomer retains functional activity, allowing the functional Sup35 protein to remain a monomer or join the prion aggregate, both of which can participate in translational termination. Mutations in the middle domain may lead to complete unfolding of Sup35 monomers (bottom), resulting in monomers that are nonfunctional. These nonfunctional proteins may stay as monomers, join the prion aggregate (as shown), form amorphous aggregates, or could be degraded.

Our studies of the [*PIN*^+^] prion suggests A503 likely has little role in fragmentation activity since the prion is maintained in Hsp104^A503S^ and Hsp104^A503V^ strains (Fig 5A and 5B). Yet our studies of the [*PSI*^+^] prion suggest that A503 has a large influence on whether the processed substrate is functional. *In vitro*, Hsp104^A503S^ and Hsp104^A503V^ can disassemble Sup35 aggregates, resulting in monomeric protein that lacks GTPase activity (Fig 6A and 6B). These results suggest that A503 mutations may change how the central pore processes substrates by completely unfolding proteins. Our *in vivo* experiments substantiate these findings since high overexpression of these variants in the presence of [*PSI*^*+*^] results in increased in stop codon readthrough (Fig 7C). Since it has been suggested that Hsp104 processes only a portion of Sup35 without disrupting the C-terminal domain [23,25,26], we speculate that the Hsp104^A503S^ and Hsp104^A503V^ variants completely unfold Sup35, including the C-terminus, to generate a non-functional protein. These unfolded monomers are unable to participate in translation termination *in vivo*, regardless if they remain monomeric, rejoin prion aggregate (as depicted in Fig 8), form amorphous aggregates or are degraded. Therefore, the [*PSI*^*+*^]-associated toxicity observed in high overexpression experiments, cytoductions, and sporulation experiments are likely due to the general loss of Sup35 GTPase function rather than the previously suggested model that variant Hsp104 could sequester soluble Sup35 into the aggregate [41]. Alternatively, these results could also support a model in which overproduction of MD variants generate amorphous aggregates or additional infectious prion particles that are able to attract more monomeric Sup35, ultimately increasing stop codon readthrough. Taken together, these results highlight the importance of the MD in Hsp104 to ensure that substrates are properly folded after processing.

Hsp104 homologs are found in both eubacteria and non-metazoan eukaryotes [78,79], and the A503 residue exhibits high conservation across fungal species [80]. While A503 mutations have been found to have potentiated activity in yeast and in heterologous systems [23,39,81–83], it is curious why these mutations do not exist in nature. Our analysis of Hsp104 functions may provide important insight. Since Hsp104^A503S^ and Hsp104^A503V^ variants are thermosensitive (S2 Fig), do not efficiently resolve stress granules (Fig 1), and exhibit toxicity with [*PSI*^+^] (Fig 4), there are likely functional evolutionary constraints on the A503 residue. Furthermore, other Hsp104-dependent prions that form from essential proteins, such as [*SWI*^*+*^] [84] could also lead to toxicity with these variants. While these Hsp104 MD variants have shown exciting promise in the disassembly of disease-related protein aggregates and can mitigate neurodegeneration in the animal CNS [39], it is important to consider the processing consequences, as the MD residue A503 can be important for generating functional processed substrates, and may control the partial threading activity required to maintain substrate function immediately after disassembly.

## Materials and methods

### Strains, plasmids, and cultivation procedures

All strains were grown at 30°C using standard media and cultivation procedures [85], except when mentioned. Rich media containing 2% dextrose (YPD) or synthetic complete media containing the required amino acids and 2% dextrose (SD), 2% galactose (Sgal) or 2% glycerol (Sglycerol) was used as indicated. Strains transformed with plasmids were maintained on synthetic complete media lacking the specific amino acid. All strains were in the 74D-694 (*Mata ade1-14 leu2-3*,*112 his3-Δ200 trp1-289 ura3-52*) background [16], unless specified. *hsp104* deletion strain in the 74D-694 background was disrupted with a *HIS3* [40] or *LEU2* cassette [86]. Cytoduction donors were derivatives of C10B-H49 (*Matα SUQ5 ade2-1 lys1-1 his3-11*,*15 leu1 kar1-1 cyhR* [*RHO+*]). Strains transformed with plasmids containing Hsp104 driven by its native promoter (pRS316HSE; series 3109–3112), a Hsp104 driven by a galactose inducible promoter (pRS416Gal; series 3073, p3288, p3075-3077), or Pab1-GFP were used as indicated. S2 Table indicates all strains, including the prion states, used in this study, and S3 Table indicates all plasmids used in this study.

For serial dilution plates, all strains were grown in liquid culture overnight to log or late log and plated at five-fold serial dilutions on YPD or the indicated selective media. A colony color assay was used to assay for [*PSI*^+^] loss [16]. 74D-694 strains, which contain an *ade1-14* nonsense allele, are red in the [*psi*^-^] state and white in the [*PSI*^+^] state when plated on nutrient rich media [YPD; 16]. YPD plates were incubated at 30°C for 2–4 days and allowed to incubate at room temperature for an additional 4–7 for color development.

### Stress granule disassembly

*hsp104Δ* strains containing the Pab1-GFP plasmid (p3285) and HSE-Hsp104 plasmids (p3109-p3112) were grown overnight to late log phase at 30°C. Cultures were heat shocked for 45 minutes at 42°C to induce stress granule formation. 3D field images were acquired every thirty minutes following heat shock for three hours, and the number of cells with Pab1-GFP granules were quantified. Images were acquired with Leica DMI 6000 fluorescent deconvolution microscope (63X, 1.4 NA) and captured with a Leica K5 camera, and images were processed with LASX software.

For 3D timelapse microscopy, indicated strains were grown overnight to late log at 30°C. Cells were then heat shocked for 45 minutes at 42°C and 200 μL of culture was immediately added to a concanavalin A-coated Ibidi 8-well glass bottom slide. Cells were incubated with 300 μL fresh SD-Ura-Trp media, and 3D images were captured every 15 minutes for 3 hours, using a 100 X, NA 1.44 objective. Images were subjected to 3D deconvolution (Media Cybernetics) and maximum projection for this publication.

### Cytoduction

Cytoduction is a method to assess transfer of cytoplasmic material from one strain to another via mating. This technique relies on one strain harboring a *kar1-1* mutation, which inhibits nuclear fusion between the donor and recipient strain [53,87]. The resulting heterokaryons have two nuclei but a mix of cytoplasmic elements, including prions, and mitochondria [53]. A cytoductant contains the recipient nucleus of interest and the inherited cytoplasm from the donor strain. Cytoduction is used to introduce prions into a *hsp104Δ* strains where the only source of Hsp104 is from the maintained plasmid.

Control cytoductions used a [*psi*^*-*^][*pin*^*-*^][*RHO*^*+*^] donor strain (M608) mated for approximately 20 hours to [*rho*°] *hsp104Δ* recipient strains (M621), each containing one of the HSE plasmids. After mating, cells were struck out on SGlycerol-Ura-His-Lys plates, where the glycerol selects against the original recipient, the -Lys and -His selects against the donor, and the -Ura selects for the plasmids. Resulting colonies are either cytoductants and heterokaryons. To determine whether colonies were cytoductants or heterokayrons, colonies were picked and pronged onto selective media to verify genetic markers. For subsequent cytoductions, [*PSI*^*+*^] (from strain D133) or [*PIN*^*+*^] (from strain D225) was donated to a *hsp104Δ* [*rho*°] strain (M621) containing one of the HSE plasmids. To assess whether [*PSI*^*+*^] is propagated and inherited by subsequent daughter cells, [*PSI*^*+*^] state was determined by growth on SD-Ura-Ade media, since the presence of the [*PSI*^+^] prion leads to suppression of the *ade1-14* allele which contains a premature UGA stop codon. To determine [*PIN*^*+*^] maintenance, all [*PIN*^*+*^] cytoductants were mated to a tester strain containing the copper inducible RNQ1-GFP plasmid (p3036) and viewed under fluorescent microscopy for cytoplasmic aggregates after 4 hours of induction with 50 μM CuSO_4_.

### Well trap assays

Cell lysates were prepared according to Sharma et al. [88]. Lysates were treated with 2% SDS sample buffer in the presence of 8% 2-mercaptoethanol. Prior to loading, samples were either untreated or boiled for 8 minutes prior to loading. Samples were run on 10% SDS-PAGE gels and subjected to standard Western blot procedures using primary polyclonal α-Rnq1 antibody (a kind gift from Elizabeth Craig, UW-Madison) and secondary monoclonal α-rabbit HRP (1:30,000).

### Tetrad and random spore analysis

[*psi*^*-*^] and strong [*PSI*^*+*^] *HSP104/hsp104Δ* heterozygous diploids were created by mating M606 and D223 containing one of the HSE plasmids. 64D-694/74D-694 diploids sporulate poorly compared to other genetic backgrounds such as W303, which exhibits 50% sporulation in eight days [57]. To increase sporulation frequencies, diploids were patched onto pre-sporulation media (0.8% yeast extract, 0.3% bacto peptone, 10% dextrose, 100 mg/L adenine sulfate (5X), 1.5% agar) and incubated at 30°C for two days. Cells were then patched onto sporulation media lacking uracil (0.98% potassium acetate, 0.05% dextrose, 0.1% yeast extract, 20 mg/L histidine (1X), 100 mg/L leucine (1X), 20 mg/L tryptophan (1X), 100 mg/L adenine sulfate (5X), 1.5% agar; personal communication from Yury Chernoff).

To determine the success of sporulation, tetrads were stained with DAPI to count the number of nuclei. Cells from the sporulation plates were fixed with glacial acetic acid and methanol for an hour, then incubated with DAPI stain (10 mg/mL) in ethanol for five minutes. Cells were pelleted and washed with water twice before imaging. Stained nuclei were visualized using a DAPI filter.

For random spore analysis, [*PSI*^*+*^] *HSP104/hsp104Δ* heterozygous diploids containing the HSE plasmids were harvested from sporulation media lacking uracil into 0.5 mL of water. Cells were washed and vortexed in water, centrifuged and resuspended in 0.5 mL water. 25 μL of 10 mg/mL Zymolyase-20T (US Biologicals) and 5 μL of 2-Mercaptoethanol were added to the resuspended cells and incubated overnight at 30°C with gentle shaking. The next day, 0.2 mL of 1.5% Nonidet P-40 (ThermoFisher Scientific) were added to the mixtures, and tubes were vortexed vigorously. Spores were centrifuged for ten minutes at 1200 g, resuspended in 0.5 mL water, then vortexed again. A hemocytometer was used to calculate the number of spores in the mixture. Approximately 100–200 spores were plated onto SD-Ura plates, and colonies were spotted onto selective media to determine spore genotype. To assess whether spores acquired suppressor mutations, haploids were passaged onto YPD plates supplemented with 5 mM guanidine hydrochloride three times, then struck out for single colony on YPD. Only colonies that reverted to red color were considered [*PSI*^+^].

### Sup35 prion disassembly and GTPase activity assays

Preformed full-length Sup35 prions were prepared as described [25,71]. Purification of Hsp104, GroEL_trap_, and other cochaperones was described [25]. Sup35 prion disassembly in the presence of purified co-chaperones Thioflavin T (ThT) assays were performed as previously described [25]. Sup35 GTPase activity hydrolysis was measured as previously described by Krzewska et al. [68].

### Stop codon readthrough using GST(UGA)DsRed intensity with methylene blue staining

pRS304 pGPD-GST(UGA)DsRed [70] was digested with EcoRV and integrated into [*psi*^*-*^] and strong [*PSI*^*+*^] strains to generate strains M655 and M659, respectively. These strains were transformed with Hsp104 galactose-inducible plasmids used in Fig 2 (p3288, p3075-77). Since we noticed that the growth of strains transformed with HSE-Hsp104^A503S^ and Hsp104^A503V^ decline after several weeks, we allowed transformed strains to age at room temperature for approximately 10 days. Transformants were pronged onto 2% dextrose media and grown for two days to ensure that strains were viable. Once grown, plates were velveted onto 2% galactose media to induce plasmid expression. This galactose media was supplemented with an extra 200 mg/L of adenine to limit red pigment accumulation formed as a byproduct of the *ade1-14* allele, which could interfere with the DsRed detection. After two days of induction on plates, cells were scraped from the plate and resuspended in water. To omit non-viable cells from our quantification, we stained dead cells with methylene blue by pipetting 1.5 μL of resuspended cells onto glass microscope slides, and adding 1.5 μL of 1X methylene blue directly onto the cells on the slide. Cell mixtures were allowed to stain for 5 minutes before 3D field images were acquired with Leica DMI 6000 fluorescent deconvolution microscope (63X, 1.4 NA) and captured with a Leica K5 camera (Texas Red filter with 600 ms of exposure was used for DsRed fluorescence; and bright field for methylene blue stains). Images were exported and analyzed using Image Pro software. For DsRed quantification, black level was set to 300 and white level to 2,000. To set the background or baseline fluorescence of each image, the mean pixel intensity of five random areas of the field that lacked cells were averaged and used for normalization in that image. To measure the raw Ds-Red signal of each cell that did not have methylene blue staining, individual cells were selected and the mean DsRed pixel intensity within the selected area of the cell was determined. For quantification purposes (shown in Figs 7B, 7C and S6A), the mean DsRed pixel intensity of individual cells was subtracted by the field background within each respective image.

## Supporting information

S1 Fig Characterization of HSE plasmids in wildtype and *hsp104Δ* strains. A) A wildtype [*psi*^-^][*pin*^-^] strain with an integrated endogenously GFP tagged Hsp104 (M248), was transformed with plasmids that contain and Hsp104 promoter (HSE) only (empty vector; EV) or the HSE promoting driving wildtype Hsp104 (Hsp104^WT^), Hsp104^A503S^, or Hsp104^A503V^ on centromeric plasmids (see materials and methods). Cultures were stained with propidium iodide (PI) and subjected to flow cytometry. To gate for dead cells by PI staining, the EV containing strain was heat-killed at 60°C for ten minutes (top left panel). Blue scatter is indicative of dead cells; black scatter is indicative of living cells. B) Quantification of living cells from panel A from three biological replicates. Data is shown the average of three trials and standard deviation. C) Endogenous Hsp104-GFP intensity (FITC-A) of the strains used in A and B were measured via flow cytometry. The vertical fixed line provides reference relative to the baseline FITC-A peak of the control Hsp104-GFP strain without a plasmid (Hsp104-GFP). Shown is representative of three trials. D-E) Wildtype strains transformed with the indicated HSE plasmid were subjected to Western blot to assess Hsp104 levels in the presence of endogenousHsp104. A representative Western blot using anti-Hsp104 and anti-actin antibodies (D) and quantified Hsp104 steady state levels normalized to actin (E). Since the plasmid provides additional Hsp104, the fold change of plasmid overexpression by the presence of Hsp104^WT^, Hsp104^A503S^, or Hsp104^A503V^ was calculated by dividing the signal of each normalized sample to the average signal of the EV control. F and G) *hsp104Δ* strains transformed with the indicated plasmid were subjected to Western blot to assess Hsp104 levels in the absence of endogenous Hsp104. Representative Western blot of the indicated plasmids in *hsp104Δ* strains (F) Quantification of Hsp104 expression in a *hsp104Δ* strain, normalized to actin for three independent Western blots (G). All samples were subjected to a Brown-Forsythe ANOVA with Dunnett’s T3 multiple comparisons test for significance.
(TIF)

S2 Fig Hsp104^A503V^ does not confer thermotolerance. A) Five-fold serial dilutions of a WT [*psi*^-^][*pin*^-^] strain transformed with indicated Hsp104 plasmids were plated on SD-Ura and incubated at indicated temperatures. Toxicity was assessed after 3 or 6 days of incubation. B) Plasmids transformed in a *hsp104Δ* strain were incubated similar to A. Toxicity was assessed after 2 days of incubation C) A *hsp104Δ* strain was transformed with plasmids (order similar to B). Cultures were treated at 37°C for 30 minutes, or additionally subjected to thermotolerance (pretreated at 37°C for 30 minutes followed by 50°C for 30 minutes) prior to plating on SD-Ura. Toxicity was assessed on the indicated days. All images are representative of two trials.
(TIF)

S3 Fig Moderate overexpression of Hsp104 variants is not toxic in the presence of strong [*PSI*^*+*^] and do not cure strong [*PSI*^*+*^]. Wildtype strong [*PSI*^*+*^][*pin*^-^] strains with indicated HSE plasmids were plated on SD-Ura (left) to assess toxicity after 3 to 6 days. The same cultures were also plated on YPD (right) to assess [*PSI*^*+*^] curing. Curing was assessed by colony color 7 days after plating on YPD. Shown is a representative of three trials.
(TIF)

S4 Fig Overexpression of Hsp104^A503S^ cures weak [*PSI*^*+*^] at similar efficiency to Hsp104^WT^. A) Galactose inducible Hsp104^WT^ in weak [*PSI*^+^][*pin*^-^] strains were grown in indicated concentrations of galactose in media lacking uracil overnight. Strains were plated on YPD to assess [*PSI*^+^] curing by colony color. B) Hsp104^WT^ and Hsp104^A503S^ weak [*PSI*^+^] strains induced with 0.1% galactose were plated for single colony and assessed for the [*PSI*^+^] state. Data includes three trials, and 600–1,000 colonies counted per trial. Quantification of weak [*PSI*^*+*^] loss in indicated strains was not significant (p = 0.0637) according to a Welch’s t-test. independent Western blots (*p≤0.011). All samples were subjected to a Welch’s t-test for significance.
(TIF)

S5 Fig Hsp104^A503S^ and Hsp104^A503V^ exhibit irregular DAPI staining in the presence of [*PSI*^*+*^]. A) Representative images of diploids with various number of DAPI stained nuclei, as well as cells with irregular DAPI staining where nuclei are poorly formed (bottom). B) *Left panel*, percentage of cells that formed complete tetrads in [*psi*^*-*^] *HSP104/ hsp104Δ* heterozygous 74D-694/64D-694 diploids containing the indicated plasmids. *Right panel*. Percentage of cells that have irregular DAPI staining. C) *Left panel*, percentage of cells that formed tetrads in strong [*PSI*^*+*^] diploids containing the indicated plasmids. *Right panel*, percentage of [*PSI*^*+*^] cells with irregular DAPI staining. Each data point is representative of 250–350 cells imaged.
(TIF)

S6 Fig [*PSI*^*+*^] cells exhibit increased stop codon readthrough compared to [*psi*^-]^ cells, regardless of which Hsp104 variant is overexpressed. A) [*psi*^*-*^][*pin*^-^] and [*PSI*^*+*^][*pin*^-^] strains integrated with a GST(UGA)DsRed reporter were transformed with the indicated galactose-inducible Hsp104 variants. Plasmids were grown on galactose-containing media for two days to drive plasmid expression. Mean DsRed pixel intensity was measured in individual cells and normalized to the mean DsRed pixel intensity of the background field. Bars represent mean±SD. Respective [*psi*^*-*^] and [*PSI*^*+*^] strains were compared using an unpaired t-test with Welch’s correction (****p≤0.0001). Approximately 25–250 cells were counted for each of three trials for a minimum total of 275 cells. B) To calculate the mean difference of DsRed intensity between [*PSI*^+^] and [*psi*-] strains with the same plasmid, average DsRed intensity of [*PSI*^+^] strains in A was subtracted from the average DsRed intensity of the corresponding [*psi*-] strain.
(TIF)

S1 Data The spreadsheet contains the raw data obtained for the Sup35 prion disassembly results through thioflavin T fluorescence (Fig 6A) and the GTPase function assay through GTPase activity (Fig 6B). The readme tab indicates the recombinant proteins used in the experiment.
(XLSX)

S2 Data The spread sheet contains the raw data obtained for the GST(UGA)DsRed assay in Fig 7. Each tab contains the data collected for each individual strain. The readme tab indicates the strains and plasmids used in Fig 7, as well as what values are contained in each column.
(XLSX)

S1 Table Trials of random sporulation. (DOCX)

S2 Table Strains (in order as appeared in text) used in this study. (DOCX)

S3 Table Plasmids (in order as appeared in text) used in this study. (DOCX)

S1 Text SUPPLEMENTAL MATERIALS AND METHODS. (DOCX)
