# Supplementary figures and images for: The middle domain of Hsp104 can ensure substrates are functional after processing
Source: PLoS Genet. 2024 Oct 3;20(10):e1011424. doi: 10.1371/journal.pgen.1011424 (PMC11478891; doi:10.1371/journal.pgen.1011424)

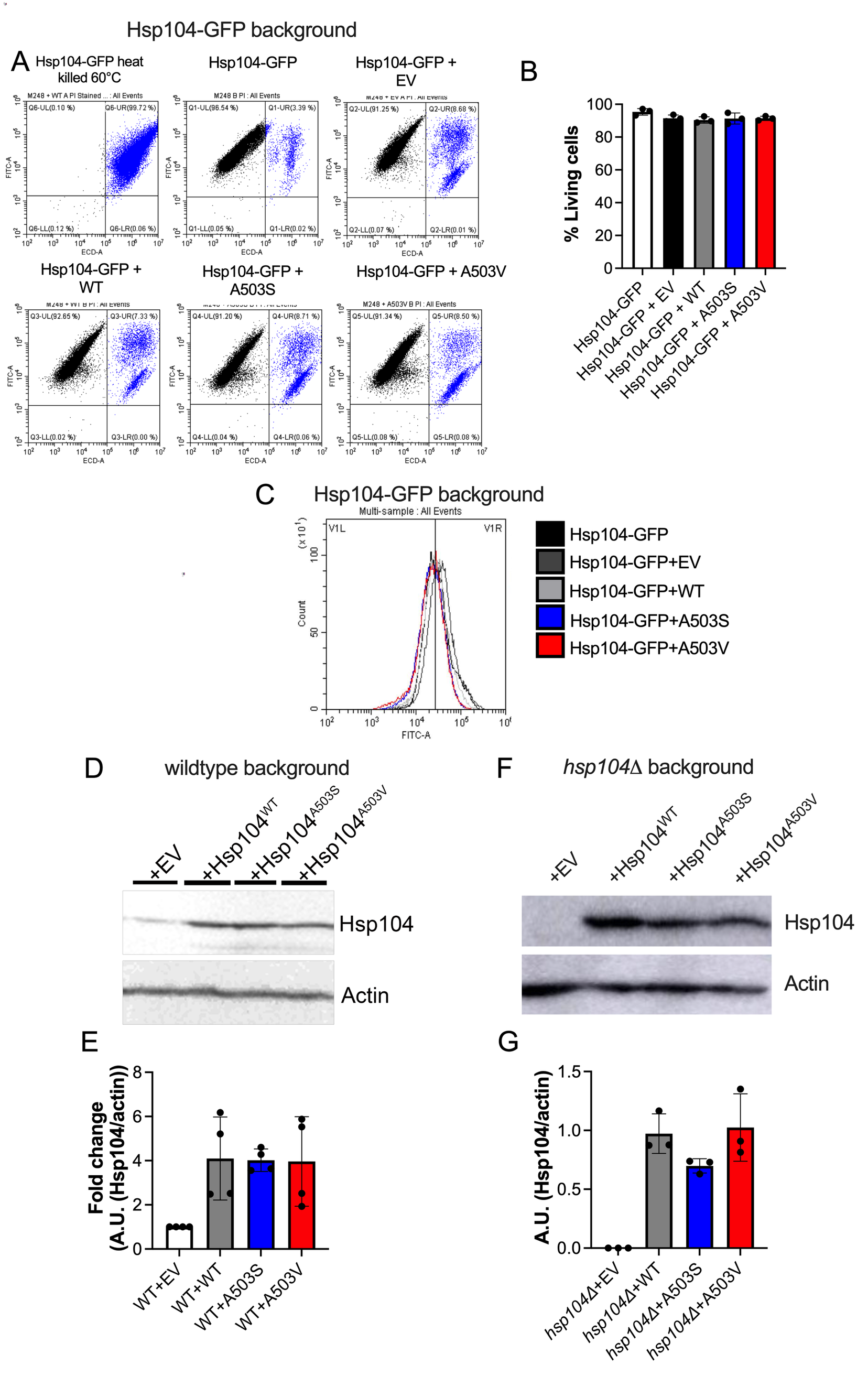

Supplement: S1 Fig — A) A wildtype [psi-][pin-] strain with an integrated endogenously GFP tagged Hsp104 (M248), was transformed with plasmids that contain and Hsp104 promoter (HSE) only (empty vector; EV) or the HSE promoting driving wildtype Hsp104 (Hsp104WT), Hsp104A503S, or Hsp104A503V on centromeric plasmids (see materials and methods). Cultures were stained with propidium iodide (PI) and subjected to flow cytometry. To gate for dead cells by PI staining, the EV containing strain was heat-killed at 60°C for ten minutes (top left panel). Blue scatter is indicative of dead cells; black scatter is indicative of living cells. B) Quantification of living cells from panel A from three biological replicates. Data is shown the average of three trials and standard deviation. C) Endogenous Hsp104-GFP intensity (FITC-A) of the strains used in A and B were measured via flow cytometry. The vertical fixed line provides reference relative to the baseline FITC-A peak of the control Hsp104-GFP strain without a plasmid (Hsp104-GFP). Shown is representative of three trials. D-E) Wildtype strains transformed with the indicated HSE plasmid were subjected to Western blot to assess Hsp104 levels in the presence of endogenousHsp104. A representative Western blot using anti-Hsp104 and anti-actin antibodies (D) and quantified Hsp104 steady state levels normalized to actin (E). Since the plasmid provides additional Hsp104, the fold change of plasmid overexpression by the presence of Hsp104WT, Hsp104A503S, or Hsp104A503V was calculated by dividing the signal of each normalized sample to the average signal of the EV control. F and G) hsp104Δ strains transformed with the indicated plasmid were subjected to Western blot to assess Hsp104 levels in the absence of endogenous Hsp104. Representative Western blot of the indicated plasmids in hsp104Δ strains (F) Quantification of Hsp104 expression in a hsp104Δ strain, normalized to actin for three independent Western blots (G). All samples were subjected to a Brown-For [file pgen.1011424.s001.tif]

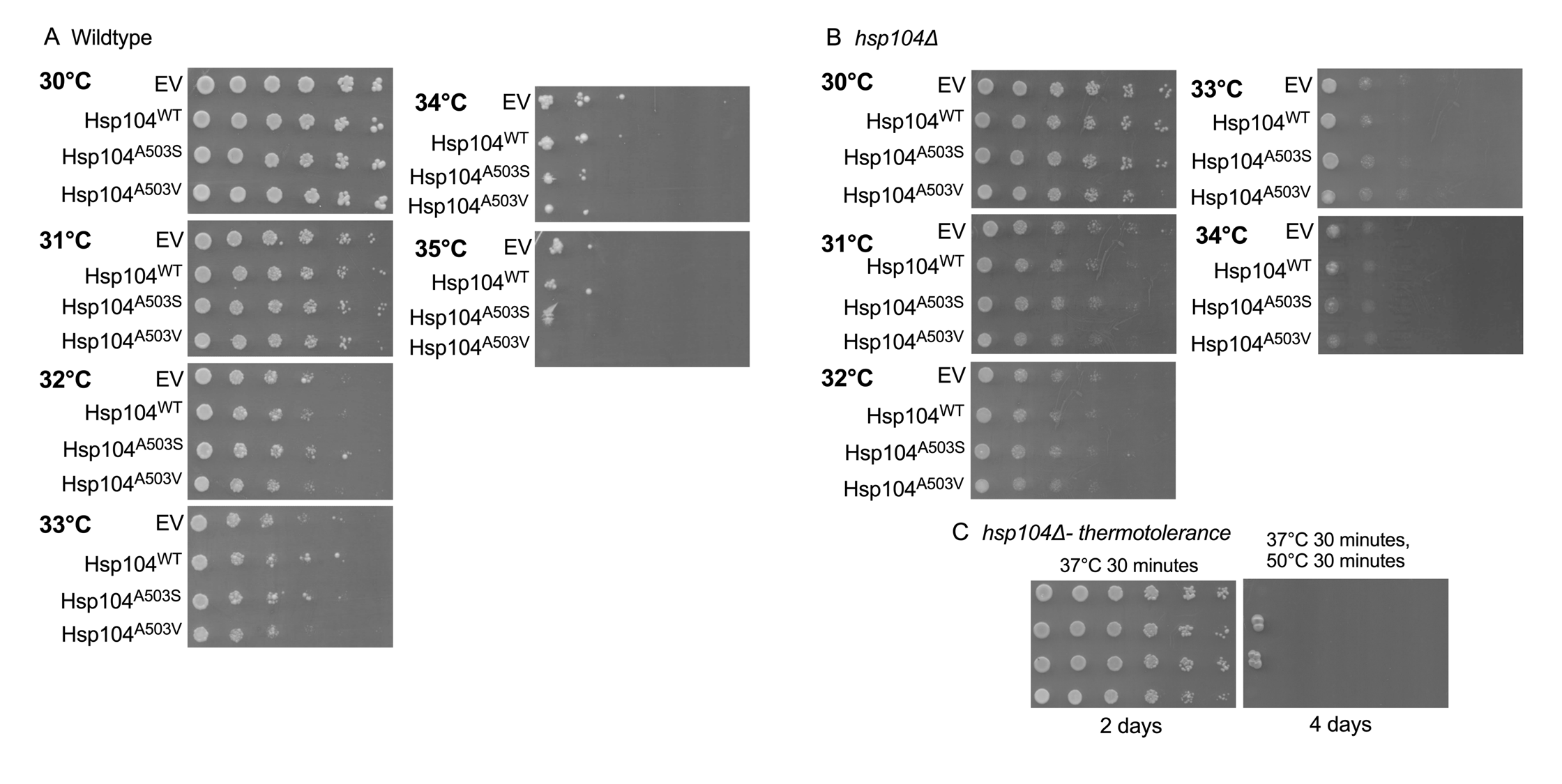

Supplement: S2 Fig — A) Five-fold serial dilutions of a WT [psi-][pin-] strain transformed with indicated Hsp104 plasmids were plated on SD-Ura and incubated at indicated temperatures. Toxicity was assessed after 3 or 6 days of incubation. B) Plasmids transformed in a hsp104Δ strain were incubated similar to A. Toxicity was assessed after 2 days of incubation C) A hsp104Δ strain was transformed with plasmids (order similar to B). Cultures were treated at 37°C for 30 minutes, or additionally subjected to thermotolerance (pretreated at 37°C for 30 minutes followed by 50°C for 30 minutes) prior to plating on SD-Ura. Toxicity was assessed on the indicated days. All images are representative of two trials. (TIF) [file pgen.1011424.s002.tif]

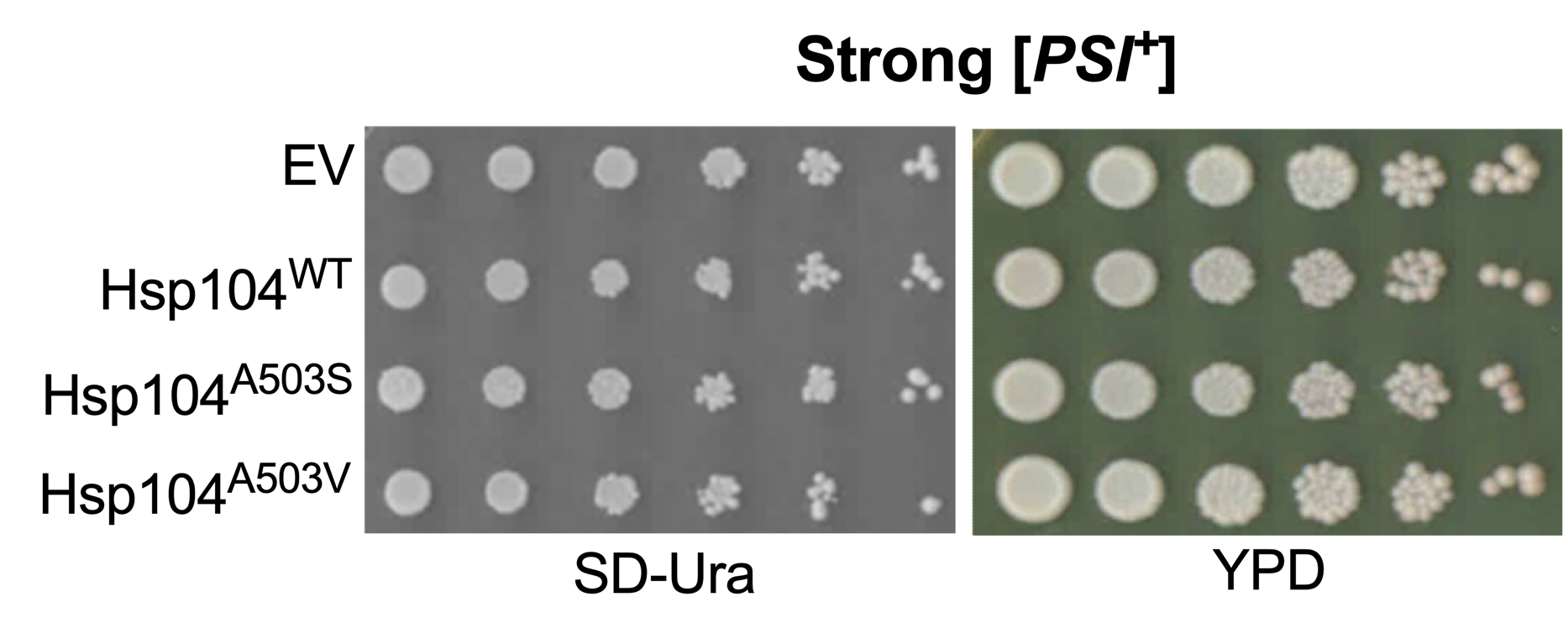

Supplement: S3 Fig — Wildtype strong [PSI+][pin-] strains with indicated HSE plasmids were plated on SD-Ura (left) to assess toxicity after 3 to 6 days. The same cultures were also plated on YPD (right) to assess [PSI+] curing. Curing was assessed by colony color 7 days after plating on YPD. Shown is a representative of three trials. (TIF) [file pgen.1011424.s003.tif]

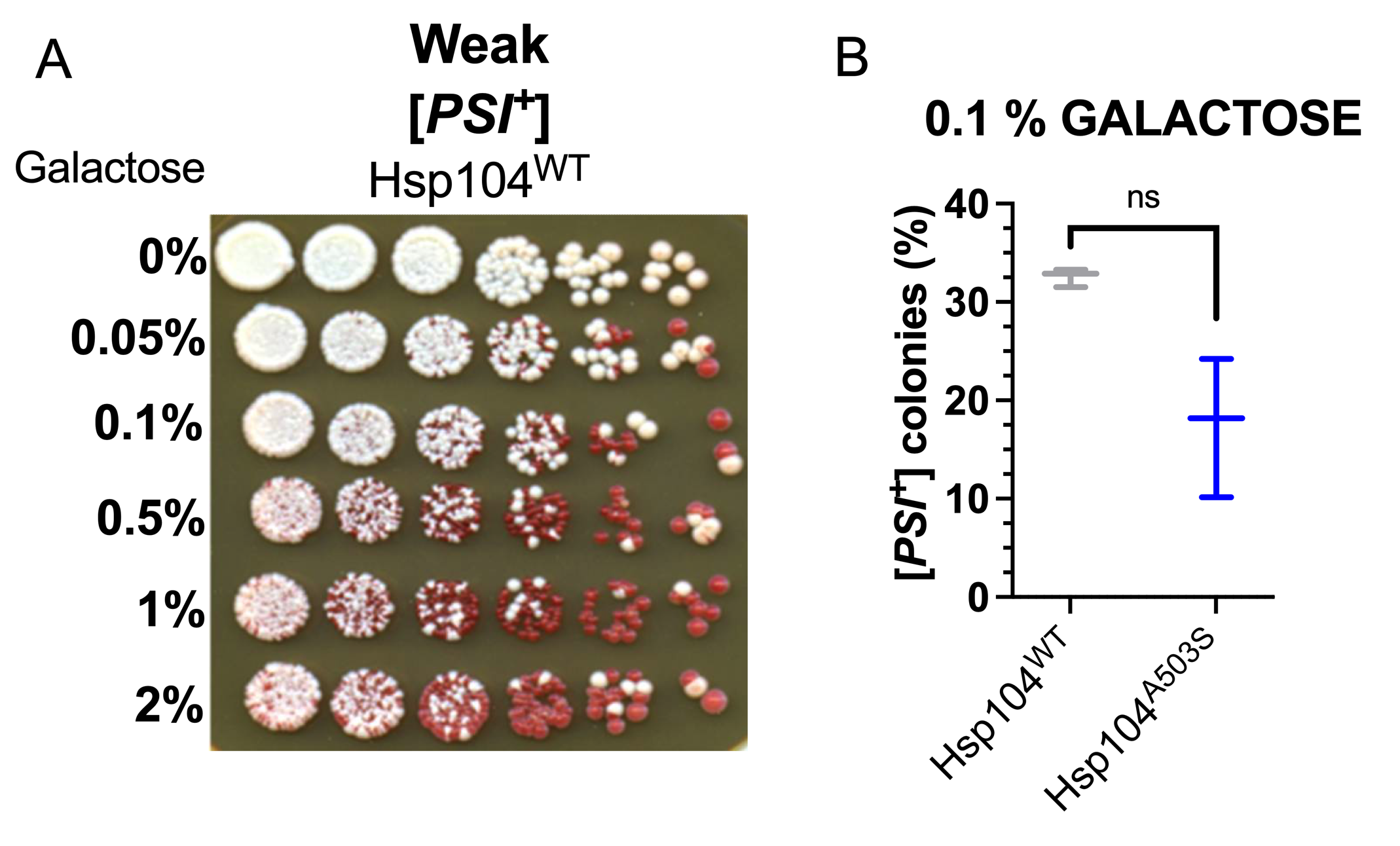

Supplement: S4 Fig — A) Galactose inducible Hsp104WT in weak [PSI+][pin-] strains were grown in indicated concentrations of galactose in media lacking uracil overnight. Strains were plated on YPD to assess [PSI+] curing by colony color. B) Hsp104WT and Hsp104A503S weak [PSI+] strains induced with 0.1% galactose were plated for single colony and assessed for the [PSI+] state. Data includes three trials, and 600–1,000 colonies counted per trial. Quantification of weak [PSI+] loss in indicated strains was not significant (p = 0.0637) according to a Welch’s t-test. independent Western blots (*p≤0.011). All samples were subjected to a Welch’s t-test for significance. (TIF) [file pgen.1011424.s004.tif]

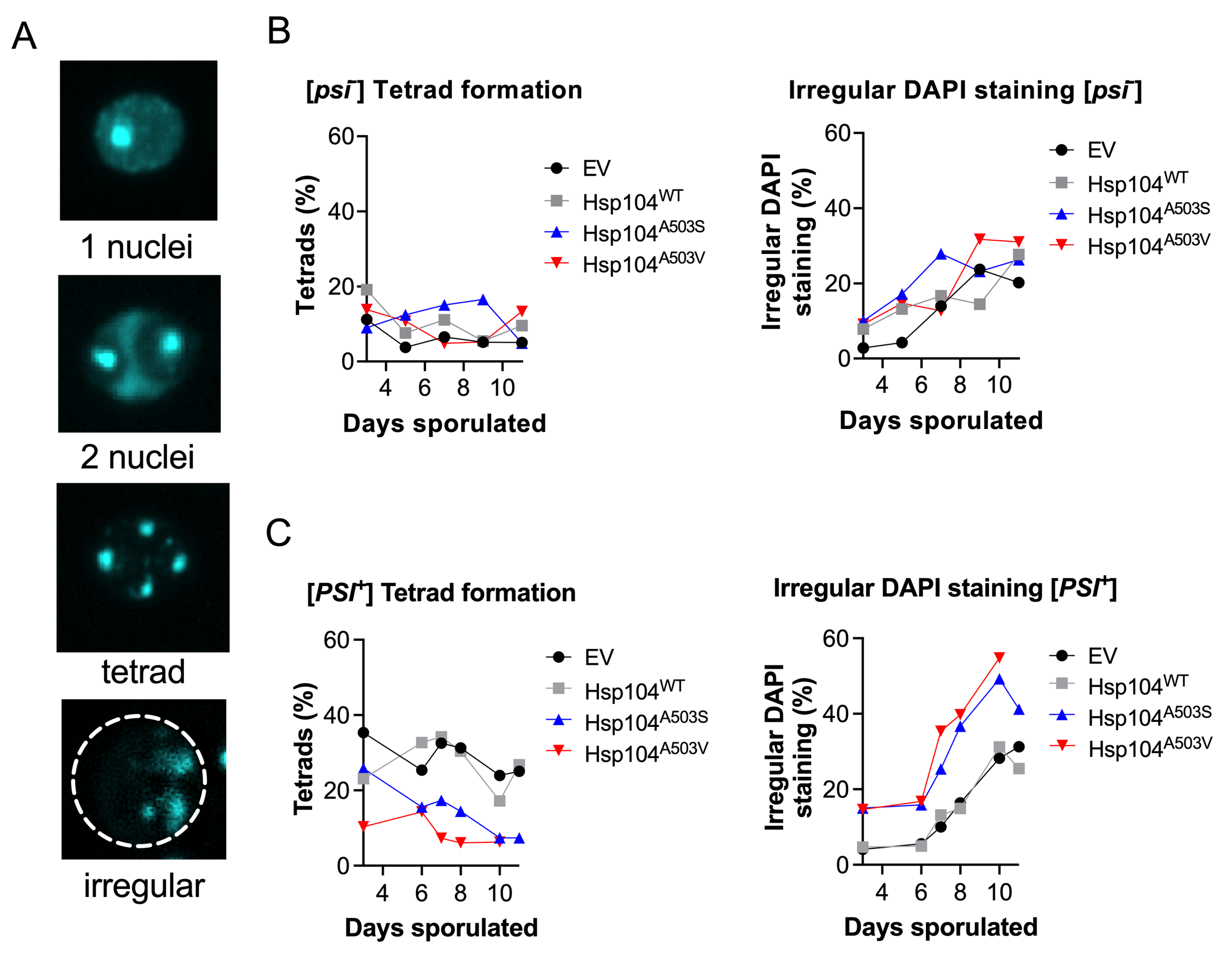

Supplement: S5 Fig — A) Representative images of diploids with various number of DAPI stained nuclei, as well as cells with irregular DAPI staining where nuclei are poorly formed (bottom). B) Left panel, percentage of cells that formed complete tetrads in [psi-] HSP104/ hsp104Δ heterozygous 74D-694/64D-694 diploids containing the indicated plasmids. Right panel. Percentage of cells that have irregular DAPI staining. C) Left panel, percentage of cells that formed tetrads in strong [PSI+] diploids containing the indicated plasmids. Right panel, percentage of [PSI+] cells with irregular DAPI staining. Each data point is representative of 250–350 cells imaged. (TIF) [file pgen.1011424.s005.tif]

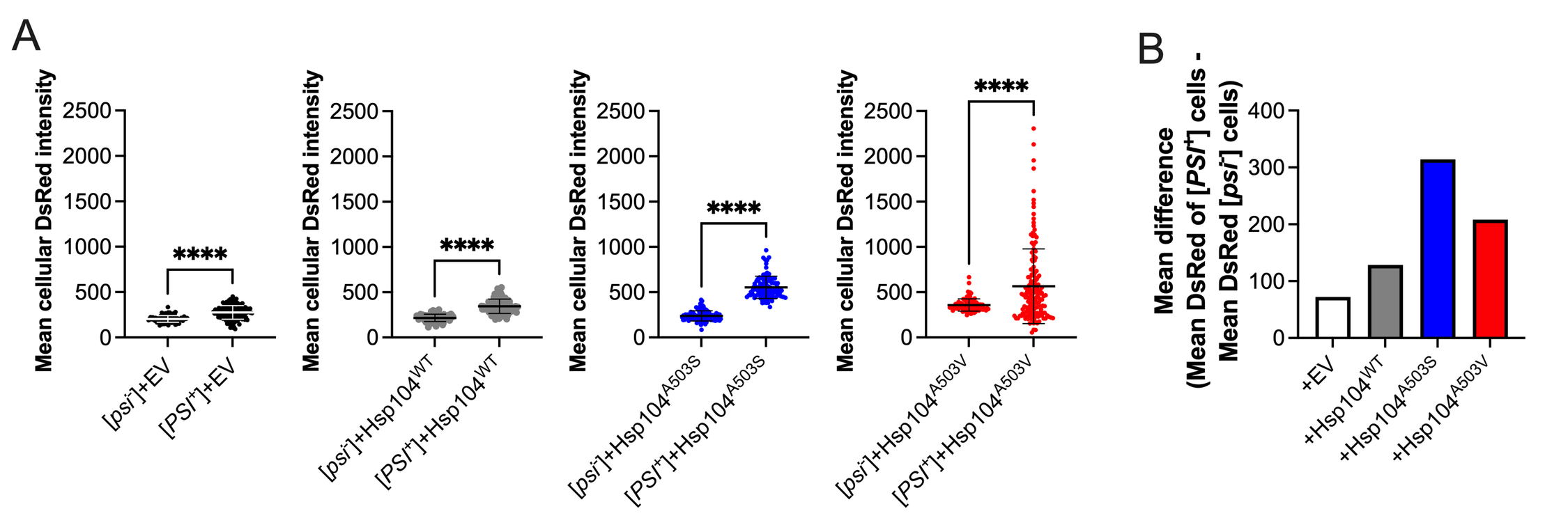

Supplement: S6 Fig — A) [psi-][pin-] and [PSI+][pin-] strains integrated with a GST(UGA)DsRed reporter were transformed with the indicated galactose-inducible Hsp104 variants. Plasmids were grown on galactose-containing media for two days to drive plasmid expression. Mean DsRed pixel intensity was measured in individual cells and normalized to the mean DsRed pixel intensity of the background field. Bars represent mean±SD. Respective [psi-] and [PSI+] strains were compared using an unpaired t-test with Welch’s correction (****p≤0.0001). Approximately 25–250 cells were counted for each of three trials for a minimum total of 275 cells. B) To calculate the mean difference of DsRed intensity between [PSI+] and [psi-] strains with the same plasmid, average DsRed intensity of [PSI+] strains in A was subtracted from the average DsRed intensity of the corresponding [psi-] strain. (TIF) [file pgen.1011424.s006.tif]
