# Supplementary material for: The middle domain of Hsp104 can ensure substrates are functional after processing
Source: PLoS Genet. 2024 Oct 3;20(10):e1011424. doi: 10.1371/journal.pgen.1011424 (PMC11478891; doi:10.1371/journal.pgen.1011424)
Supplement: S1 Table — (DOCX) [file pgen.1011424.s009.docx]

**S1 Table.** Trials of random sporulation

| **Trial 1.** |  |  |  |  |
| --- | --- | --- | --- | --- |
| Strain | **EV** | **Hsp104^WT^** | **Hsp104^A503S^** | **Hsp104^A503V^** |
| Total spores obtained | 24 | 13 | 16 | 0 |
| % [*PSI^+^*] | 0 | 72.9 | 0 | N/A |
| % [*psi^-^*] | 100 | 27.1 | 100 | N/A |
| **Trial 2.** |  |  |  |  |
| Strain | **EV** | **Hsp104^WT^** | **Hsp104^A503S^** | **Hsp104^A503V^** |
| Total spores obtained | 81 | 72 | 271* | 0 |
| % [*PSI^+^*] | 0 | 76.4 | 0 | N/A |
| % [*psi^-^*] | 100 | 23.6 | 100 | N/A |

Spores referred to in the table are *hsp104Δ* that contain the plasmid. See Table 1 for combined data. *Due to results from the first trial, we over-picked Hsp104^A503S^ colonies.
