# Supplementary material for: The middle domain of Hsp104 can ensure substrates are functional after processing
Source: PLoS Genet. 2024 Oct 3;20(10):e1011424. doi: 10.1371/journal.pgen.1011424 (PMC11478891; doi:10.1371/journal.pgen.1011424)
Supplement: S2 Table — (DOCX) [file pgen.1011424.s010.docx]

**Supplemental Table 2.** Strains (in order as appeared in text) used in this study.

| Strain | Genotype | Name | Reference and Figure |
| --- | --- | --- | --- |
| M248 | *Mat****α*** *ade1-14 his3-200 trp1-289 ura3-52 leu2-3,112 HSP104GFP::KANMX6*  [*psi^-^*][*pin*^-^] | Hsp104-GFP 74D-694 | [1] (Figure S1) |
| D223 | *Mat****a*** *ade1-14 leu2-3, 112 his3-delta200 trp1-289 ura3-52 hsp104::LEU2*  [*psi^-^*][*pin^-^*] | *hsp104Δ* 74D-694 | [2] (Figure S2, S4, S5, 1; Table 1, S1) |
| D230 | *Mat****a*** *ade1-14 ura3-52 leu2-3,112 trp1-289 his3-200*  [*psi^-^*][*pin^-^*] | [*psi*^+^][*pin*^+^] 74D-694 | [3]  (Figure S2, S5, 5; Table 1, S1) |
| D114 | *Mat****a*** *ade1-14 ura3-52 leu2-3,112 trp1-289 his3-200*  Strong [*PSI^+^*][*pin^-^*] | Strong [*PSI*^+^] 74D-694 | Kind gift from Susan Liebman (original strain name: L1763) (Figure S3, 2) |
| D112 | *Mat****a*** *ade1-14 ura3-52 leu2-3,112 trp1-289 his3-200*  Weak [*PSI^+^*][*pin^-^*] | Weak [*PSI*^+^] 74D-694 | Kind gift from Susan Liebman (original strain name: [4] (Figure 2, 3) |
| M608 | *Mat****α*** *SUQ5 ade2-1 lys1-1 his3-11,15 leu1 kar1-1 ura3::KanMX4* [*psi^-^*][*pin^-^*][*RHO*+] | [*psi^-^*][*pin^-^*] Donor strain C10-H49a | [5] (Figure 4) |
| D225 | *Mat****α*** *SUQ5 ade2-1 lys1-1 his3-11,15 leu1 kar1-1 ura3::KanMX4* [*psi^-^*] High [*PIN^+^*][*RHO*+] | [*psi^-^*] [*PIN^+^*]  Donor strain C10-H49a | [6] (Figure 4) |
| D133 | *Mat****α*** *SUQ5 ade2-1 lys1-1 his3-11,15 leu1 kar1-1 cyhR* Strong [*PSI^+^*][*pin^-^*][*RHO*+] | [*PSI^+^*][*pin^-^*]  Donor strain C10-H49 | [7] (Figure 4) |
| M621 | *Mat****a*** *ade1-14 ura3-52 leu2-3,112 trp1-289 his3-200 hsp104::HIS3*  [*psi^-^*][*pin^-^*][*rho^0^*] | *hsp104Δ* 74D-694 | This study  (Figure 4) |
| M606 | *Mat****α*** *ade1-14, ura3-52, leu2-3,112, trp1-289, lys9-A21* Strong [*PSI^+^*][*pin*^-^] | Strong [*PSI*^+^] 64D-694 | This study  (Figure S5; Table 1, S1) |
| D233 | *Mat****a*** *ade1-14 ura3-52 leu2-3,112 trp1-289 his3-200* [*psi-*] High [*PIN^+^*] | High [*PIN^+^*] 74D-694 | (Derkatch et al., 1997)  (Figure 5) |
| M666 | *Mat****a*** *ade1-14 ura3-52 leu2-3,112 trp1-289 his3-200 hsp104::HIS3* [*psi*^-^]*+* p3109 (EV)  cytoduced w/ High [*PIN^+^*] | *hsp104Δ* 74D-694 | This study  (Figure 5) |
| M667 | *Mat****a*** *ade1-14 ura3-52 leu2-3,112 trp1-289 his3-200 hsp104::HIS3* [*psi*^-^] *+* p3110 (WT-Hsp104)  cytoduced w/ High [*PIN^+^*] | [*PIN^+^*] cytoductant Hsp104^WT^ 74D-694 | This study  (Figure 5) |
| M668 | *Mat****a*** *ade1-14 ura3-52 leu2-3,112 trp1-289 his3-200 hsp104::HIS3* [*psi*^-^] *+* p3112 (Hsp104-A503S) cytoduced w/ High [*PIN^+^*] | [*PIN^+^*] cytoductant Hsp104^A503S^ 74D-694 | This study  (Figure 5) |
| M669 | *Mat****a*** *ade1-14 ura3-52 leu2-3,112 trp1-289 his3-200 hsp104::HIS3* [*psi*^-^] *+* p3111 (Hsp104-A503V) cytoduced w/ High [*PIN^+^*] | [*PIN^+^*] cytoductant Hsp104^A503V^ 74D-694 | This study  (Figure 5) |
| M655 | *Mat****a*** *ade1-14 ura3-52 leu2-3,112 trp1-289 his3-200* [*psi-*][*pin-*] GST(UGA)::DsRed *(TRP1)* | [*psi^-^*] integrated with GST(UGA)DsRed 74D-694 | This study  (Figure 7; S6) |
| M659 | *Mat****a*** *ade1-14 ura3-52 leu2-3,112 trp1-289 his3-200* Strong [*PSI*^+^][*pin-*] GST(UGA)::DsRed *(TRP1)* | Strong [*PSI*^+^] integrated with GST(UGA)DsRed 74D-694 | This study  (Figure 7; S6) |

References

1. Klaips CL, Hochstrasser ML, Langlois CR, Serio TR. Spatial quality control bypasses cell-based limitations on proteostasis to promote prion curing. eLife. 2014;3. Epub 2014/12/10. doi: 10.7554/eLife.04288. PubMed PMID: 25490068; PubMed Central PMCID: PMCPMC4270096.

2. Zhou P, Derkatch IL, Liebman SW. The relationship between visible intracellular aggregates that appear after overexpression of Sup35 and the yeast prion-like elements [PSI(+)] and [PIN(+)]. Mol Microbiol. 2001;39(1):37-46. Epub 2000/12/21. doi: mmi2224 [pii]. PubMed PMID: 11123686.

3. Chernoff YO, Lindquist SL, Ono B, Inge-Vechtomov SG, Liebman SW. Role of the chaperone protein Hsp104 in propagation of the yeast prion-like factor [psi+]. Science. 1995;268(5212):880-4. Epub 1995/05/12. PubMed PMID: 7754373.

4. Derkatch IL, Chernoff YO, Kushnirov VV, Inge-Vechtomov SG, Liebman SW. Genesis and variability of [PSI] prion factors in Saccharomyces cerevisiae. Genetics. 1996;144(4):1375-86. Epub 1996/12/01. PubMed PMID: 8978027; PubMed Central PMCID: PMC1207691.

5. Kochneva-Pervukhova NV, Poznyakovski AI, Smirnov VN, Ter-Avanesyan MD. C-terminal truncation of the Sup35 protein increases the frequency of de novo generation of a prion-based [PSI+] determinant in Saccharomyces cerevisiae. Curr Genet. 1998;34(2):146-51. Epub 1998/09/02. PubMed PMID: 9724418.

6. Bradley ME, Liebman SW. Destabilizing interactions among [PSI(+)] and [PIN(+)] yeast prion variants. Genetics. 2003;165(4):1675-85. Epub 2004/01/06. PubMed PMID: 14704158; PubMed Central PMCID: PMC1462903.

7. Manogaran AL, Kirkland KT, Liebman SW. An engineered nonsense URA3 allele provides a versatile system to detect the presence, absence and appearance of the [PSI+] prion in Saccharomyces cerevisiae. Yeast. 2006;23(2):141-7. Epub 2006/02/24. doi: 10.1002/yea.1341. PubMed PMID: 16491470; PubMed Central PMCID: PMC2600413.
