# Supplementary material for: The middle domain of Hsp104 can ensure substrates are functional after processing
Source: PLoS Genet. 2024 Oct 3;20(10):e1011424. doi: 10.1371/journal.pgen.1011424 (PMC11478891; doi:10.1371/journal.pgen.1011424)
Supplement: S3 Table — (DOCX) [file pgen.1011424.s011.docx]

**Supplemental Table 3.** Plasmids (in order as appeared in text) used in this study.

| Plasmid Number | Description | Yeast Marker | Reference |
| --- | --- | --- | --- |
| p3109 | pRS316HSE | *URA3* | [1] |
| p3110 | pRS316HSE Hsp104 WT | *URA3* | [1] |
| p3112 | pRS316HSE Hsp104A503S | *URA3* | [2] |
| p3111 | pRS316HSE Hsp104A503V | *URA3* | [1] |
| p3285 | PAB1-GFP | *TRP1* | [3] |
| p3073 | pRS416Gal EV | *URA3* | [1] |
| p3288 | pRS416Gal EV | *URA3* | This study (Figure 2, 6) |
| p3075 | pRS416Gal-WTHsp104 | *URA3* | [1] |
| p3077 | pRS416Gal-HSP104A503S | *URA3* | [1] |
| p3076 | pRS416Gal-HSP104A503V | *URA3* | [1] |
| p3189 | pGal-Sup35C | *TRP1* | [4] |
| p3036 | RNQ1-GFP | *LEU2* | [5] |
| p3054 | pRS304 GPD GST(UGA)DsRed | *TRP1* | [6] |

References

1. Jackrel ME, DeSantis ME, Martinez BA, Castellano LM, Stewart RM, Caldwell KA, et al. Potentiated Hsp104 variants antagonize diverse proteotoxic misfolding events. Cell. 2014;156(1-2):170-82. doi: 10.1016/j.cell.2013.11.047. PubMed PMID: 24439375; PubMed Central PMCID: PMCPMC3909490.

2. Howard MK, Sohn BS, von Borcke J, Xu A, Jackrel ME. Functional analysis of proposed substrate-binding residues of Hsp104. PLoS One. 2020;15(3):e0230198. Epub 2020/03/11. doi: 10.1371/journal.pone.0230198. PubMed PMID: 32155221; PubMed Central PMCID: PMCPMC7064214.

3. Brengues M, Parker R. Accumulation of polyadenylated mRNA, Pab1p, eIF4E, and eIF4G with P-bodies in Saccharomyces cerevisiae. Mol Biol Cell. 2007;18(7):2592-602. Epub 2007/05/04. doi: 10.1091/mbc.e06-12-1149. PubMed PMID: 17475768; PubMed Central PMCID: PMCPMC1924816.

4. Vishveshwara N, Bradley ME, Liebman SW. Sequestration of essential proteins causes prion associated toxicity in yeast. Mol Microbiol. 2009;73(6):1101-14. PubMed PMID: 19682262.

5. Sondheimer N, Lindquist S. Rnq1: an epigenetic modifier of protein function in yeast. Mol Cell. 2000;5(1):163-72. PubMed PMID: 10678178.

6. Satpute-Krishnan P, Serio TR. Prion protein remodelling confers an immediate phenotypic switch. Nature. 2005;437(7056):262-5. PubMed PMID: 16148935.
