# Supplementary material for: The middle domain of Hsp104 can ensure substrates are functional after processing
Source: PLoS Genet. 2024 Oct 3;20(10):e1011424. doi: 10.1371/journal.pgen.1011424 (PMC11478891; doi:10.1371/journal.pgen.1011424)
Supplement: S1 Text — (DOCX) [file pgen.1011424.s012.docx]

**SUPPLEMENTAL MATERIALS AND METHODS:**

*Flow cytometry*

A Hsp104-GFP strain (M248) containing centromeric plasmids with Hsp104 driven by *HSP104* promoters (HSE) were grown to saturation overnight in synthetic SD-Ura media. Flow cytometry was performed using a Cytoflex Flow Cytometer (Beckman Coulter) to measure the amount of dead cells, stained with propidium iodide (PI; ECD-A filter), and GFP fluorescence intensity (FITC-A filter). For PI staining, 400 μL of cells were pelleted and resuspended in 1X PBS pH 7.2. 2 μL of PI (2 mg/mL) was added, and cells were incubated on ice in the dark for 15 minutes. 50,000-100,000 cells were counted per sample. Histograms and scatterplots were generated using CytExpert Software.

*Hsp104 steady state levels*

Western blotting was used to determine Hsp104 steady state levels in wildtype and *hsp104Δ* strains using primary polyclonal α-Hsp104 antibody (1:10,000; Thermofisher, PA1-024), secondary monoclonal α-rabbit HRP (1:10,000), and subject to chemiluminescence. Blots were washed and subjected to primary monoclonal α-actin (1:1,000), and secondary α-mouse AP (1:10,000) as a loading control. Hsp104 steady state levels were determined by quantifying Hsp104 signal using ImageJ and normalized levels to actin.

*Thermotolerance and chronic heat stress assays*

Thermotolerance assays were performed as described in Sanchez and Lindquist (1990) [1]. A *hsp104Δ* strain containing the Hsp104 promoter plasmids were grown to late log phase overnight at 30°C in SD-Ura media. Cultures were normalized to an OD_600_ of 0.6 and incubated at 37°C for 30 min, followed by an additional heat treatment at 50°C for 30 minutes. Cultures were serially diluted fivefold and spotted on SD-Ura media. The plates were incubated at 30°C for 3 days. Chronic heat stress assays were performed by serial diluting strains on selective media and incubating strains at 30°C, 31°C, 32°C, 33°C, 34°C, and 35°C for 2-6 days.


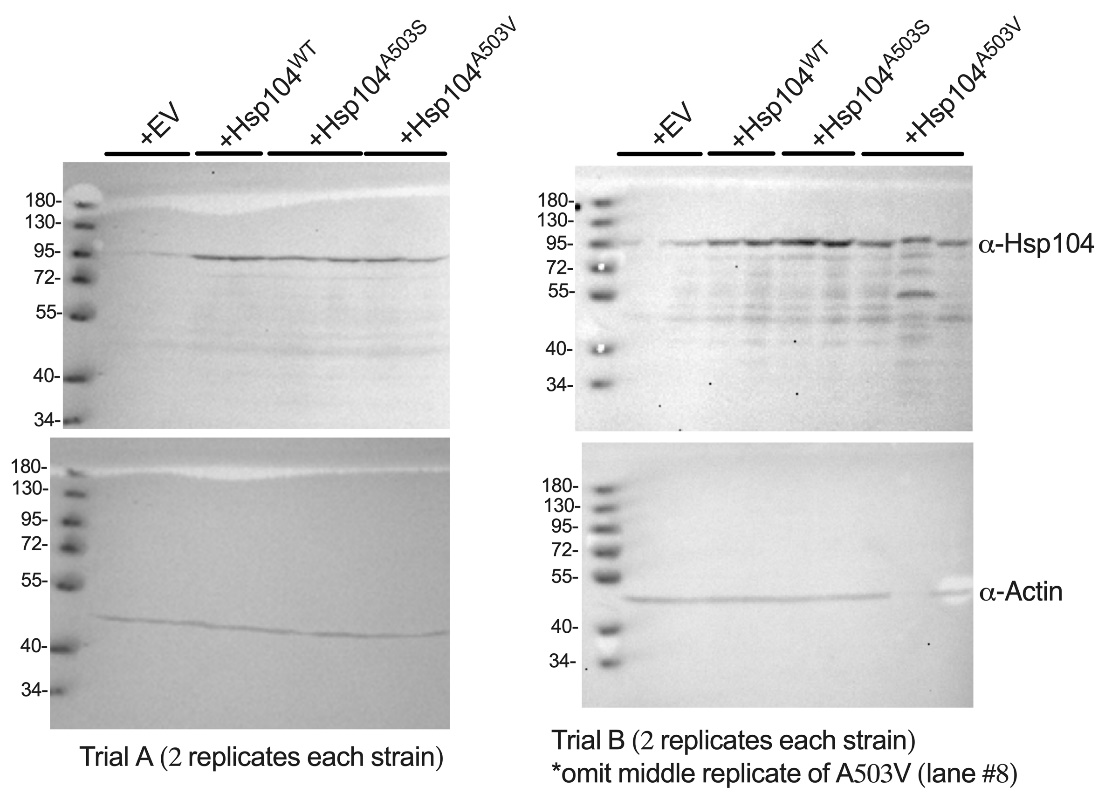


Supplemental Raw Blots of HSE-driven, ‘moderate overexpression’ Hsp104 plasmid overexpression (p3109-3112) in a wildtype strain. Relevant to S1D Fig. Note that for trial B, lane 8 was omitted from Hsp104^A503V^ quantification due to a loading error.


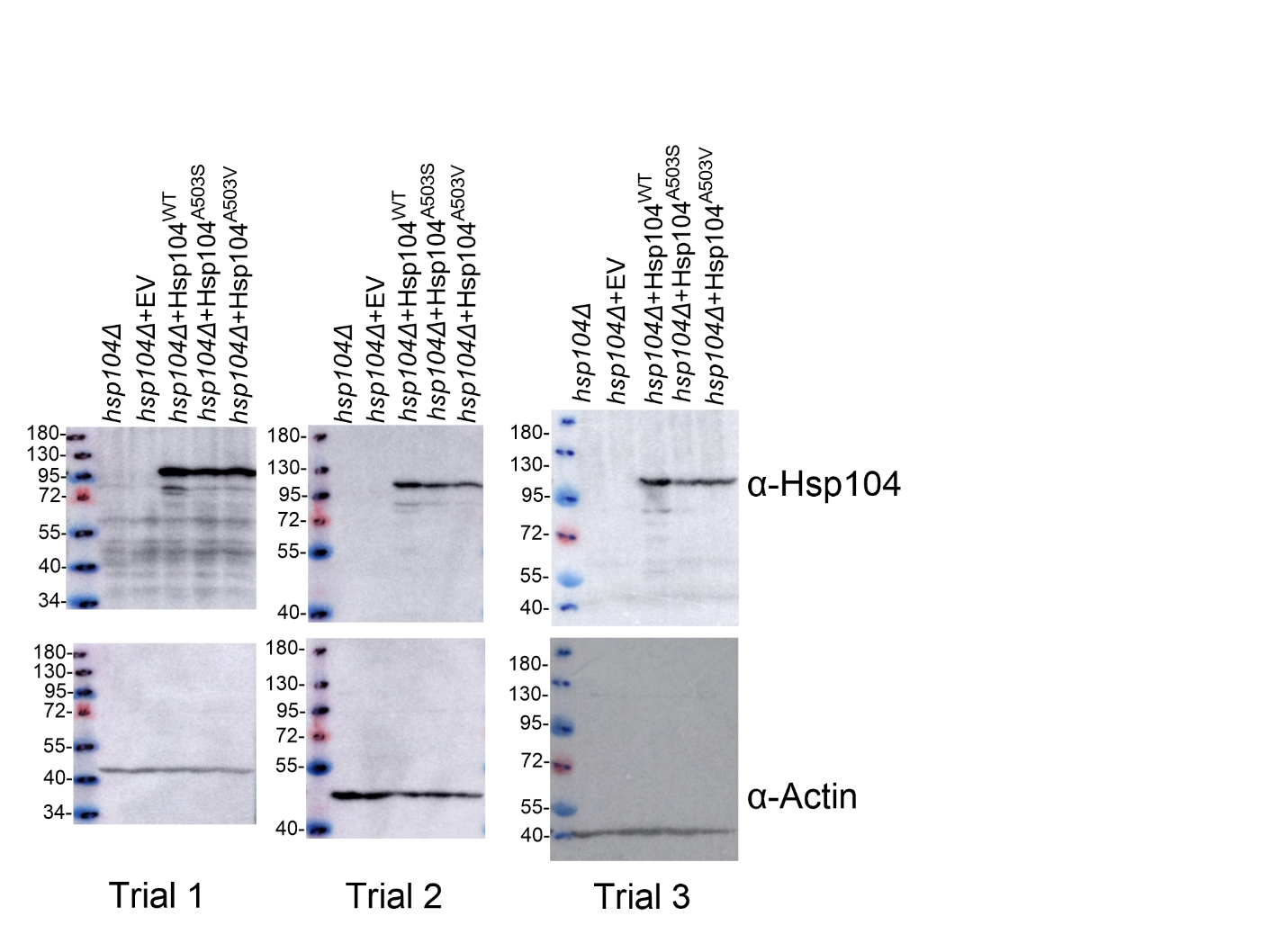


Supplemental Raw Blots of HSE-driven, ‘moderate overexpression’ Hsp104 plasmid overexpression (p3109-3112) in *hsp104Δ*. Relevant to S1F Fig.


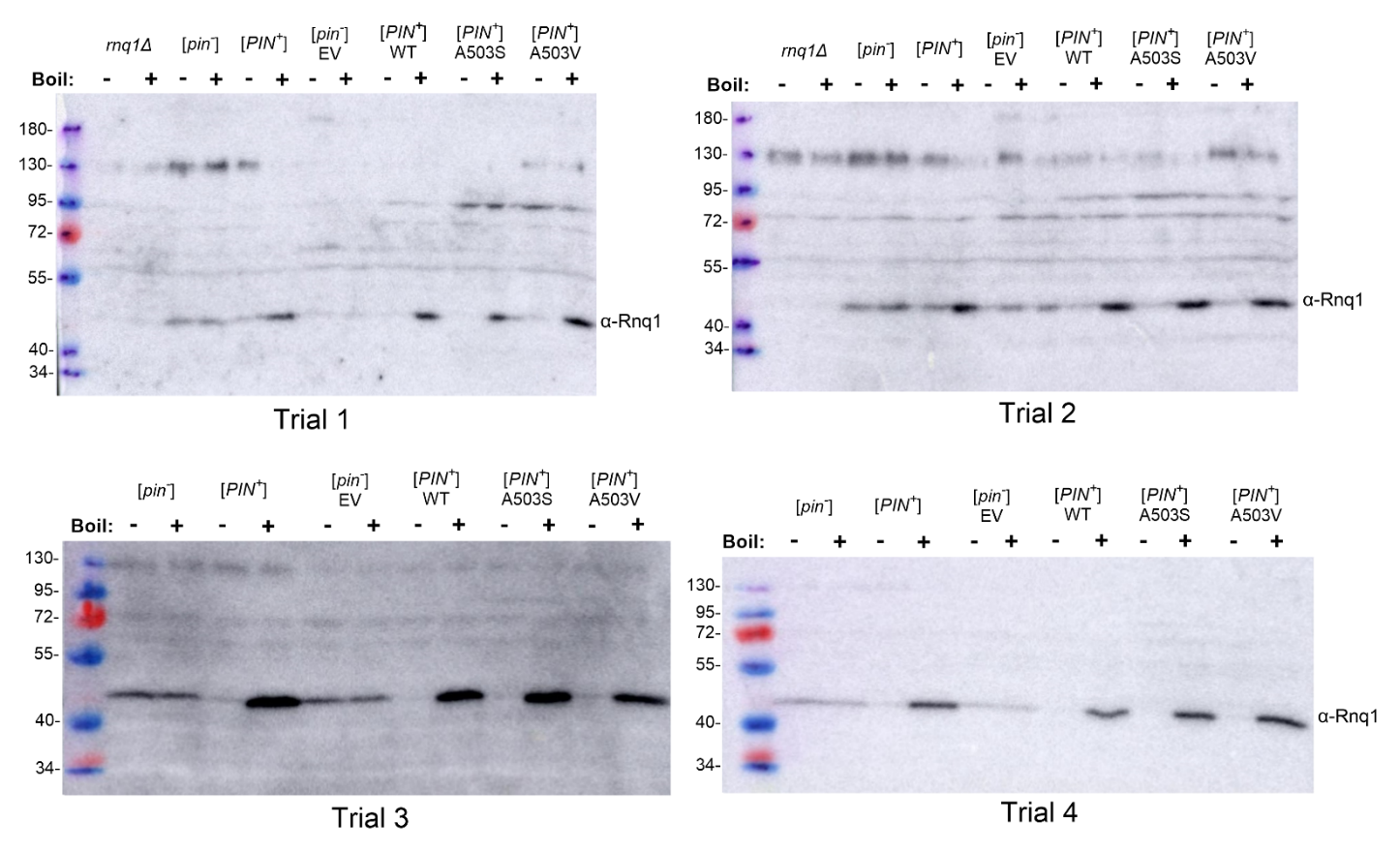


Supplemental Raw Blots of well trap assays of Rnq1. Relevant to Fig 5C.

Literature Cited

1. Sanchez, Y., and S.L. Lindquist. 1990. HSP104 required for induced thermotolerance. *Science*. 248:1112-1115.
